# Polyandry and sperm competition in two traumatically inseminating species of Strepsiptera (Insecta)

**DOI:** 10.1038/s41598-024-61109-z

**Published:** 2024-05-07

**Authors:** Kenny Jandausch, Nico Wanjura, Hermes Escalona, Manuela Sann, Rolf G. Beutel, Hans Pohl, Oliver Niehuis

**Affiliations:** 1 Entomology Group, Institut für Zoologie und Evolutionsforschung, Friedrich-Schiller-Universität Jena, Erbertstraße 1, 07743 Jena, Germany; 2 Department of Evolutionary Biology and Ecology, University of Freiburg, Hauptstraße 1, 79104 Freiburg, Germany; 3 Institute for Anatomie I, Jena University Hospital, Teichgraben 7, 07743 Jena, Germany; 4 Australian National Insect Collection, CSIRO, GPO Box 1700, Canberra, ACT 2601 Australia; 5 Institute for Biology (190T), University of Hohenheim, Garbenstraße 30, 70599 Stuttgart, Germany

**Keywords:** Monandrous, Microsatellites, Mate guarding, Paternity tests, *Stylops ovinae*, *Xenos vesparum*, Sperm competition, Evolution, Molecular biology, Zoology

## Abstract

Polyandry, the practice of females mating with multiple males, is a strategy found in many insect groups. Whether it increases the likelihood of receiving beneficial genes from male partners and other potential benefits for females is controversial. Strepsiptera are generally considered monandrous, but in a few species females have been observed copulating serially with multiple males. Here we show that the offspring of a single female can have multiple fathers in two Strepsiptera species: *Stylops ovinae* (Stylopidae) and *Xenos vesparum* (Xenidae). We studied female polyandry in natural populations of these two species by analysis of polymorphic microsatellite loci. Our results showed that several fathers can be involved in both species, in some cases up to four. Mating experiments with *S. ovinae* have shown that the first male to mates with a given female contributes to a higher percentage of the offspring than subsequent males. In *X. vesparum*, however, we found no significant correlation between mating duration and offspring contribution. The prolonged copulation observed in *S. ovinae* may have the advantage of reducing competition with sperm from other males. Our results show that monandry may not be the general pattern of reproduction in the insect order Strepsiptera.

## Introduction

Copulation with multiple mating partners is a strategy that can confer fitness advantages over intraspecific competitors^1^. This type of promiscuous sexual reproduction has long been considered to be the domain of males^2,3^, which have a higher variance in their reproductive success than females. This phenomenon is summarised by the Bateman principle. However, females may also use polyandry, the mating of a female with several males, as a mating strategy, which is widespread throughout the animal kingdom (e.g., Refs.^4–8^). This strategy has advantages and disadvantages for females. Parker and Birkhead^9^ list the advantages of this mating strategy to include an increased number of fertilised eggs, increased genetic diversity of the offspring, and the general allowance of postcopulatory mate choice. The same authors mention an increased risk of predation as a disadvantage, since mating is often a vulnerable period^9^. In his monograph on sperm competition, Simmons^10^ summarised five factors that could explain the evolution of polyandry: (i) sperm replenishment—females remate to replenish sperm reserves depleted by previous ovipositions, to replace inviable sperm, or to otherwise ensure fertility; (ii) material benefits—females remate to acquire resources controlled by males, such as nesting sites, food resources, or protection from conspecifics and/or heterospecifics; (iii) genetic benefits—females replace sperm from previous matings with sperm from a genetically superior mate, encourage sperm competition to ensure fertilization by high quality sperm, or ensure genetic diversity in offspring; (iv) convenience: females remate to minimize the cost of harassment by males; and (v) correlated evolution—females remate due to correlated response to sexual selection on multiple mating males. However, the idea that polyandrous females have a genetic advantage has been questioned by Slatyer et al.^11^, who found only weak support for this in a comprehensive meta-analysis of experimental studies.

Polyandric behavior creates a competitive environment for sperm from different males within the body of the inseminated female after copulation^1,10,12,13^. Two key factors are currently considered to generate this competitive environment: (1) the temporal and (2) the spatial simultaneous presence of sperm from different males in the genital tract, or more generally, inside the female’s body^1,10^. This overlap can be strongly influenced by the morphology of the female reproductive apparatus. For example, the structure, function, and number of the spermathecae can strongly influence sperm competition^14^. Such adaptations allow females to select preferred sperm for fertilization. As a result, males may have evolved counter-strategies in their post-copulatory behavior^1^. For example, dragonflies using specialised structures to remove of sperm from a previous mating partner^15^ and some spiders use a mating plug to close the genital opening of a female after mating^16^ or prolong the duration of copulation known as mate guarding^1,17^. Sperm competition can thus strengthen sexual selection^1^. Whether polyandry and sperm competition may have contributed to the evolution of the bizarre species of the little-known insect order Strepsiptera remains unclear.

The endoparasitic holometabolous order Strepsiptera comprises ca. 600 described species and is characterised by numerous derived features in both sexes at all life stages^18–21^. All species of Strepsiptera show extreme sexual dimorphism. Male Strepsiptera are free-living; the only function of their short adult life span of a few hours is to find females and to mate with them. The females are obligate endoparasites of other insects, in which they remain during most of their larval development and as adults, except for species of the Mengenillidae, whose adult females are free-living. The inability of females to actively prevent conspecific males from copulating increases the likelihood that multiple males will contribute to the fertilization of a female’s eggs (i.e., polyandry)^20,22^. However, there is only anecdotal evidence for polyandry in Strepsiptera, and it is based on a few random observations of multiple males copulating serially with the same female^23–25^. Whether these observations are representative of the behavior of the species studied or even of the entire insect order is unclear, as is the question of whether multiple males also fertilize the eggs of a given female. We conducted field and controlled laboratory experiments in combination with DNA paternity testing to answer these questions. These answers, in turn, could explain the peculiar reproductive behavior of some Strepsiptera, in which a first mating male has a longer copulation duration than a second mating male^20^.

**Figure 1:**
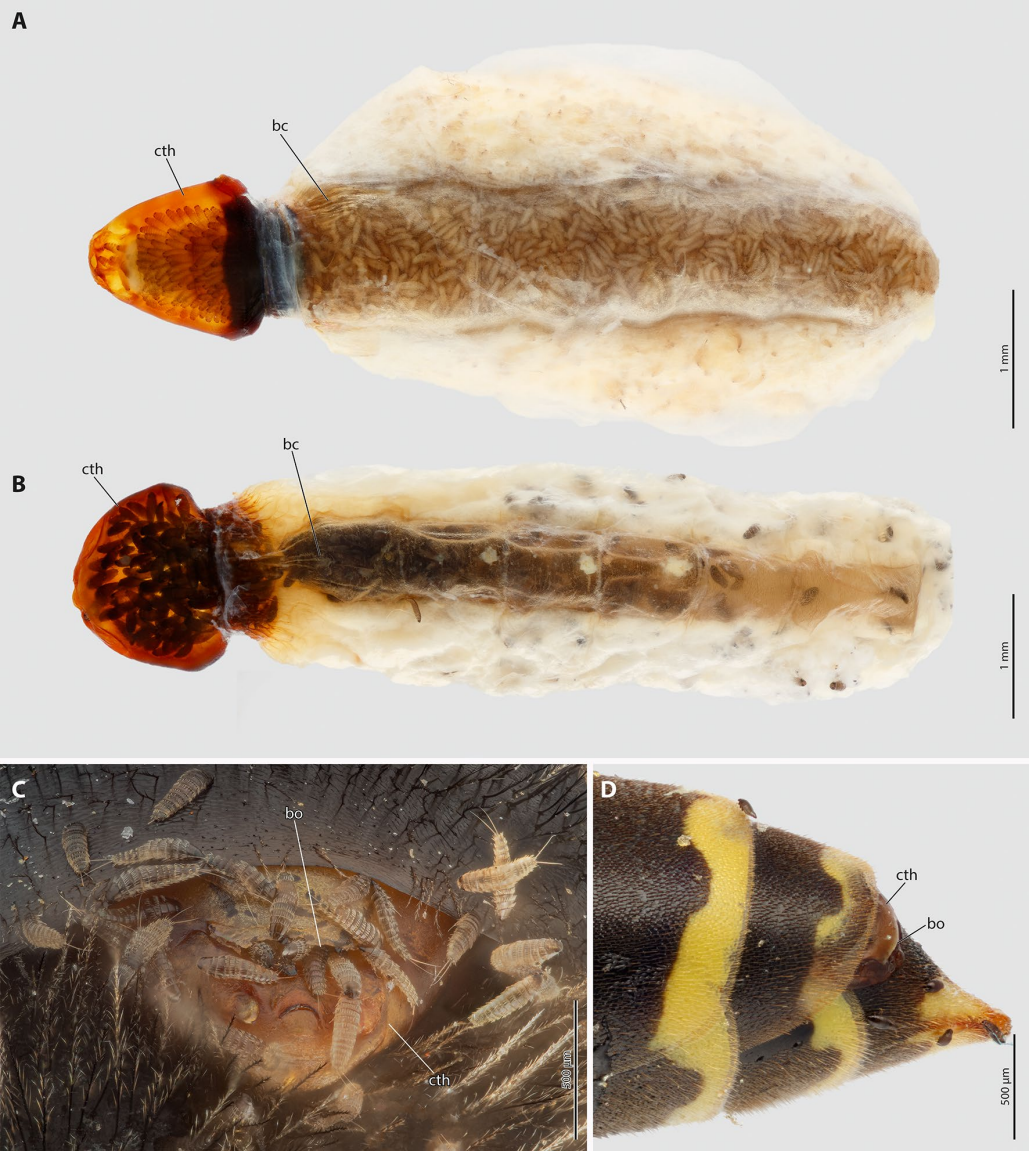
*Stylops ovinae* and *Xenos vesparum*. (**A**,**B**) Cephalothorax and brood canal of females of *S. ovinae* (**A**) and *X. vesparum* (**B**) filled with primary larvae, ventral view. (**C**) The bee *Andrena vaga* with primary larvae of *S. ovinae* emerging from the birth opening and dispersing. (**D**) The wasp *Polistes dominula* with emerging *X. vesparum* larvae. *bc* brood canal, *bo* birth opening, *cth* cephalothorax.

The Strepsiptera species whose mating behavior we study are *Stylops ovinae* (Stylopidae) and *Xenos vesparum* (Xenidae). Both belong to the clade Stylopidia, which is characterized by the use of pterygote insect hosts and by the fact that adult females remain inside the host’s body. The large sack-shaped posterior body of the female resides within the abdomen of the host, while the sclerotized cephalothorax is exposed^18,19,26,27^. In the majority of Strepsiptera (i.e., Stylopiformia, Stylopidia excl. Corioxenidae), a birth opening is present on the ventral side of the cephalothorax, between the head and the prosternum. This birth opening is connected to the hemocoel via the birth organs in the abdomen by a brood canal and allows the primary larvae to leave the female (Fig. 1). In species of Stylopiformia, the birth opening additionally serves as the site where the penis penetrates the female’s cuticle during traumatic insemination.

Kathirithamby et al.^28^ considered Strepsiptera to be monandric. This idea is based on several behavioral observations, especially in species of the family Xenidae^29,30^ and Stylopidae^31,32^: for example, *S. ovinae* (= *S. melittae* auct.) females reduce the production of a sex pheromone that attracts conspecific males immediately after the first copulation. This reduces the chance of being fertilized by multiple males. However, females do not appear to completely stop releasing their sex pheromone^33^. At the same time, there are numerous scattered hints in the literature that indicate the possible occurrence of polyandry in Strepsiptera. For example, Silvestri^25^ and Riek^24^ reported that females of species in the family Halictophagidae to had mated with multiple males and that these females remained attractive to other potential mates. Similar evidence for the occurrence of polyandry was provided by Kirkpatrick^23^ in one species of Corioxenidae. The most recent and detailed evidence for the occurrence of polyandry in Strepsiptera was presented by Peinert et al.^20^ who conducted controlled laboratory experiments showing that female *S. ovinae* mate serially with multiple males and that these females remained attractive for a prolonged period of time (up to 2 h) after the first copulation.

Strepsiptera are thought to be traumatically inseminated^21^ and this insect order therefore serves as a prime example of this mode of copulation. Traumatic inseminating (TI) is defined as a copulation method in which the copulatory organ is pierced directly through the body integument of the female, allowing the male's sperm to enter directly into the female's body cavity. This bizarre mode of copulation, combined with the endoparasitic lifestyle of the female Strepsiptera, which thus cannot prevent copulation with males and may have no precopulatory influence on mate choice, makes polyandry likely. The occurrence of TI itself is probably triggered by sperm competition, as this allows males to bypass the female genitalia and thereby also sperm previously deposited by other males^34–37^. Whether Strepsiptera females would benefit from polyandry is unclear. Given the large number of eggs produced by female Strepsiptera (e.g., *Eoxenos laboulbenei* (Mengenilldae): ca. 1400^38^; *S. ovinae*: ca. 29,000, unpublished data by H. Pohl & H. Stark; *Stichotrema dallatorreanum* (Myrmecolacidae): up to 750,000^39^), polyandry could help females to get the necessary amount of sperm to fertilize all their eggs (sperm replenishment hypothesis). However, this interpretation remains speculative, as it is difficult to gather supporting evidence due to various challenges associated with handling and rearing Strepsiptera.

Polyandry puts strong selective pressure on males to be the first to mate with a given female and to prevent or delay fertilization of the eggs by other males. The absence of sperm storage structures and ovaries in adult females most likely precludes post-copulatory mate choice, as sperm is released directly into the hemolymph by males. Structures such as the spermalege described by Carayon^40^ in bed bugs, which is thought to allow cryptic mate choice with the help of hemocytes^41,42^, are also not found in Strepsiptera. One way for males to reduce sperm competition is to extend the duration of their copulation and/or to guard their mating partner. Both strategies would delay access to the female by other males. Consistent with this idea, Peinert et al.^20^ found not only exceptionally long copulation durations of a first mating male of *S. ovinae* (up to 34 min), but also significantly shorter copulation durations of each subsequent mating male. In comparison, the reported copulation duration of *X. vesparum* is 5–15 s^43^, that of *Elenchus tenuicornis* (Elenchidae) 1–3 s^44^, and that of *Corioxenos antestiae* (Corioxenidae) a few seconds to 1 min, rarely 5 min^23^. Based on these observations, Peinert et al.^20^ discussed the possibility that a first male that mates with a given female in *S. ovinae* guards the female, thereby reducing sperm competition with other males. A longer copulation duration could alternatively or additionally allow the male to inject more sperm into the female. However, we think this is unlikely: if the total amount of sperm, rather than the order of insemination, primarily determines how many eggs are fertilized by a male (‘fair raffle’ scenario sensu Parker^45^), the copulation duration of subsequent males should not differ drastically from that of the first male. We consider it more likely that *S. ovinae* engages in mate guarding and that this strategy has evolved in this species due to the highly synchronized hatching of its males. This creates a highly competitive environment, with several males often competing for a given female (Supplementary Movie 2^20^). If there is a high probability that a male's sperm will compete with that of other males for fertilization of a given female’s eggs, and if injected sperm will result in a timely fertilization of that female's eggs, then males should benefit from restricting access to that female. If the chance of a given female being mated by multiple males during the short lifetime of the first mating male is low, a male might benefit more from searching for additional females than from guarding a female. Note that males typically live less than six hours.

In the present investigation, we use experimental behavioral assays in combination with paternity tests based on newly established microsatellite markers to assess whether multiple mating in Strepsiptera actually results in the fertilization of a female's eggs by multiple males. With all the necessary tools in place, we will additionally address the question of whether the extended copulation duration of the first copulating male in *S. ovinae* reduces the chances of subsequent copulating males to inseminate the eggs of the same female, compared to species that do not exhibit mate guarding behavior. We used *S. ovinae* and *X. vesparum* for our tests. While *S. ovinae* parasitizes only in the grey-backed mining bee (*Andrena vaga*, Andrenidae) and the ashy mining bee (*A. cineraria*)^46^, *X. vesparum* parasitizes several species of the paper wasp genus *Polistes*, primarily *Polistes dominula*^47^, but also in *Ropalidia* (Vespidae). Both species offer the advantage that they have a high infestation rate and are far easier to collect in sufficient numbers for the experiments. This is mainly due to the lifestyle of their host species. *A. vaga* can form nest aggregations of up to several thousand individuals, while the eusocial *P. dominula* is very common in settlements in Central Europe. Furthermore, these two species are suitable because they can be kept in the laboratory with little effort in order to obtain the necessary first instar larvae of the parasites for the paternity experiments. Males of *S. ovinae* hatch in synchronised masses over a period of only a few of days in late winter/early spring^31,33,48,49^. In contrast, the hatching period of *X. vesparum* under natural conditions lasts at least one month, from mid-July to mid-August^50^, and can therefore be considered asynchronous. Mass emergence of males is likely not unique to *S. ovinae* and may occur in other species under certain circumstances. Jandausch et al.^22^ attracted over 100 conspecific males to unmated *X. vesparum* females during a single day, with some males even simultaneously approaching the cage containing the stylopized paper wasps. Masses of males have also been observed in *Mengenilla moldryzki* (Mengenillidae) in Tunisia^51^.

## Results

### Polyandry in field-collected Strepsiptera

#### Stylops ovinae

Of the ten pooled samples of 50 larvae each, seven had more than four different alleles at least one locus, indicating the involvement of more than one father in the generation of a female's offspring (Table 1). The number of alleles (i.e., nine) in one particular pool of primary larvae (S08) suggests at least four fathers. Thus, polyandry clearly occurs in *Stylops ovinae* and appears to be common in the population studied.

**Table 1:** Number of alleles and estimated number of fathers in batches of 50 larvae produced by different field-collected *Stylops ovinae* females. Allele counts greater than four, indicating polyandry, are highlighted in bold.

ID	Number of alleles detected per microsatellite locus				Minimum number of fathers
	So_A	So_B	So_D	So_G	
S01	4	1	4	3	1
S02	2	1	**7**	2	**3**
S03	3	2	**6**	3	**2**
S04	4	4	**6**	3	**2**
S05	3	2	4	3	1
S06	3	1	**6**	2	**2**
S07	2	0	4	3	1
S08	**5**	3	**9**	2	**4**
S09	3	4	**6**	4	**2**
S10	3	3	**7**	3	**3**

#### Xenos vesparum

Among the ten pooled samples of 50 larvae each, one (X07) had more than four different alleles at one locus, indicating the involvement of more than one father in the generation of a female's offspring (Table 2). Thus, polyandry clearly occurs in *X. vesparum*, but does not appear to be common in the population studied.

**Table 2:** Number of alleles and estimated number of fathers of batches in 50 larvae produced by different field-collected *Xenos vesparum* females. Allele counts greater than four, indicating polyandry, are highlighted in bold.

ID	Number of alleles detected per microsatellite locus				Minimum number of fathers
	Xv_D	Xv_E	Xv_F	Xv_H	
X01	3	3	3	1	1
X02	4	2	3	2	1
X03	4	3	3	1	1
X04	3	3	3	2	1
X05	3	2	2	1	1
X06	3	1	3	3	1
X07	4	**5**	2	2	**2**
X08	3	3	3	2	1
X09	3	2	3	3	1
X10	3	2	4	2	1

### Polyandry in laboratory conditions

#### Stylops ovinae

**Figure 2:**
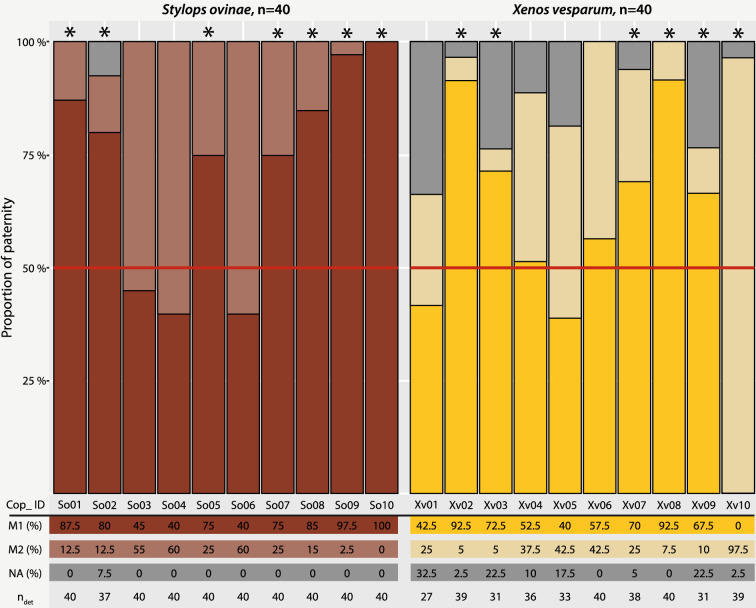
Genetic contribution of two males mating with the same female to the female’s offspring. Bar graphs show the percentage of offspring in each copulation experiment sampled (ten per species). The proportions of *Stylops ovinae* are shown in red, those of *Xenos vesparum* in yellow. The red line marks the equal distribution of 50%. Copulation experiments marked with an asterisk show a statistically significant deviation from an equal contribution of both fathers to the offspring of the female (χ^2^-test: χ^2^ = 8.53–38.011; df = 1; p < 0.004). *Cop_ID* experimental ID of the copulation experiment, *M1* proportion of offspring from the male that copulated first, *M2* proportion of offspring from the male that copulated second, *NA* not assignable; *n* number of larvae sampled, *n*_*det*_ number of larvae with known paternity.

We were able to infer the paternity of 397 of the 400 genotyped primary larvae (Fig. 2, Supplementary Table S8). The electropherograms of the remaining three primary larvae had no detectable signal. The offspring genotypes indicate that both males contributed genetically in nine out of ten experiments. Only one mating experiment (So10; n = 40) involved only a single male, the one that mated first. In seven of the ten mating experiments, the genetic contribution of the two males is statistically skewed, with the first male being a father more often than expected by chance: So01 (n = 40), So02 (n = 37), So05 (n = 40), So07 (n = 40), So08 (n = 40), So09 (n = 40), and So10 (n = 40) (χ^2^-test: χ^2^ = 10.0–40.0; df = 1; p < 0.0016).

#### Xenos vesparum

We inferred the paternity for 354 of the 400 genotyped primary larvae (Fig. 2, Supplementary Table S9). The allelic composition of the remaining 46 primary larvae did not allow a clear identification of a father. The paternity data showed that both males contributed genetically to the offspring in all but one of the experiments. Only in one mating experiment (Xv10, n = 39) was a single male identified as the father, in this case the one that mated second. The genetic contribution of the two males was statistically skewed in five of the ten mating experiments, with the first male being a father more often than expected by chance: Xv02 (n = 39), Xv03 (n = 31), Xv07 (n = 38), Xv08 (n = 40), and Xv09 (n = 31) (χ^2^-test: χ^2^ = 8.53–31.41; df = 1; p < 0.004). Only in mating experiment Xv10 (n = 39) was the second male more successful than expected by chance (χ^2^-test: χ^2^ = 38.011; df = 1; p = 7.034e−10). In contrast to males of *S. ovinae*, *X. vesparum* males often make multiple copulation attempts before copulating with a given female. We tested whether the number of copulation attempts differed between the first and second mating male, but did not find a statistically significant difference (Mann–Whitney U test: n = 18, W = 125.5, p-value = 0.2353).

### Effect of copulation duration of the first male on the paternity success of the two copulating males

#### Stylops ovinae

**Figure 3:**
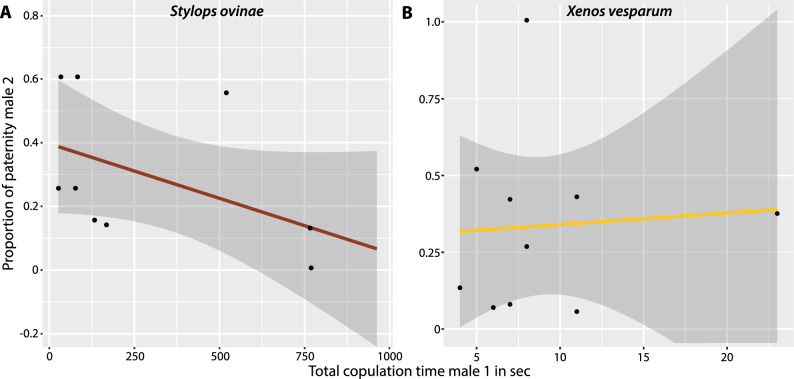
Correlation between the total copulation duration of the first male and the relative genetic contribution to the offspring of the second male in *Stylops ovinae* (**A**) and *Xenos vesparum* (**B**). Regression line in red (*S. ovinae*) and yellow (*X. vesparum*). The shape of the 95% confidence interval at is shown in dark grey.

We found a statistically significant negative correlation between the copulation duration of the first male and the paternity success of the second male (generalized linear model: n = 10; p = 0.000061; Fig. 3A). The negative correlation remained statistically significant after removing the data from experiment So10, in which the second male did not genetically contribute to the offspring (generalized linear model: n = 9; p = 0.00193). We found no effect of the copulation duration of the second male on its paternity success (generalized linear model: n = 10; p = 0.168).

#### Xenos vesparum

We found no statistically significant correlation between the copulation duration of the first male and the paternity success of the second male (generalized linear model: n = 10; p = 0.774; Fig. 3B). This result was not affected when we removed the data from experiment Xv10, in which the first male did not appear to contribute genetically to the offspring (generalized linear model: n = 9; p = 0.456441). However, we found a statistically significant negative correlation between the copulation duration of the second male and the contribution of the first male to a female's offspring (generalized linear model: n = 10; p < 0.000487).

## Discussion

Peinert et al.^20^ found that *S. ovinae* females were sequentially mated by multiple males in laboratory experiments. However, it remained unclear whether males that subsequently copulate also contribute genetically to a female's offspring. Our parental testing has shown that the offspring of a female can indeed have multiple fathers. They also showed that polyandry can be common in nature in Strepsiptera: ca. 70% in the studied population of *S. ovinae*. Peinert et al.^20^ observed that the first copulation in *S. ovinae* is significantly longer than subsequent copulations with other males and tentatively interpreted this conspicuous behavior as mate guarding. The results of our paternity tests confirm this, as we found a statistically significant negative correlation between the copulation duration of the first mating male and the fertilisation success of a second male: the first mating male benefits from prolonging his copulation with a given female in time. A positive correlation between the duration of copulation and fertilisation success has also been described in other polyandrous organisms, such as the scorpion fly *Panorpa cognata*^52^, the nursery web spider *Pisaura mirabili*^17,53^*,* and the ensign fly *Sepsis cynipsea*^54^. Our interpretation of the results also seems plausible from a morphological point of view: Strepsipteran females lack sperm storage structures. Traumatic insemination results in an immediate contact of the sperm with the eggs floating freely in the hemolymph. Since there are no known mechanisms or morphological structures that would allow cryptic sperm choice of female Strepsiptera, the first mating male gains a high degree of control over how many eggs are fertilized by his own sperm. The second mating male cannot gain an advantage by mating with females that have long since been fertilized because egg production ends with the end of postembryonic development. This makes eggs a limited resource, and the second mating male has no access to eggs that the first male did not at least have the potential to fertilize.

In contrast to the experiments of Dallai et al.^29^, which suggested that males of *X. vesparum* are not attracted to previously mated females, our experiments show that females of this species remain attractive to males for an extended period of time, even if these females have previously been fertilized by another male. In our laboratory experiments, males mated with females up to 20 min after the females were mated by another male (Supplementary Table S5, Xv01); and paternity tests show that both males contribute genetically to the offspring (Fig. 2). In nature, however, the proportion of females whose eggs are fertilized by multiple males is likely to be lower, 10% in the case of the population we studied.

Males of *X. vesparum* hatch asynchronously over a period of approximately 1 month^50^. The chances of two males competing for access to a given female at the same time are therefore likely to be significantly lower in this species than in *S. ovinae*. It is therefore unlikely that *X. vesparum* males benefit from mate guarding, and males that invest their short lifespan in searching for a second mate may have higher overall reproductive success. Consistent with these considerations, we found no evidence for a longer copulation duration of the first mating male compared to the second. We also found that the copulation duration of the first male did not correlate with the relative genetic contribution of the male to the female's offspring, even when the female was subsequently mated by a second male. While we found that the copulation duration of the second mating male negatively affects the relative genetic contribution of the first male to a female's offspring in laboratory mating experiments, we expect this pattern to be largely irrelevant under natural conditions. This is because our laboratory experiments allowed males to copulate with one female in rapid succession. This is unlikely to occur in nature, as *X. vesparum* males emerge over a long period of time, with little chance of aggregation. A possible explanation for the copulation duration of the second mating male affecting the genetic contribution of the first male to the offspring could be that the sperm of both males compete directly with each other for a limited number of eggs, with a longer copulation allowing a male to transfer more sperm ('fair raffle' scenario sensu Parker^1^). However, we consider this scenario as unlikely for several reasons, including the fact that insemination begins at the onset of the copulation, and a short mating duration is sufficient for males to transfer enough sperm to fertilize all of the eggs^31,49^.

We found no statistically significant difference in the duration of the first and of the second copulation in our experiments (Supplementary Table S4), which were less than 10 s on average. Therefore, we suggest that *X. vesparum* males, in contrast to *S. ovinae* males^20^, are not able to judge whether a female is already mated*.* We found that *X. vesparum* males make multiple attempts to mate with a given female. While we interpreted most of these copulations as failed attempts, they may represent true matings with sperm transfer. Such behavior has been shown to allow males of some species to transfer more sperm (e.g., Refs.^55–57^). However, we consider this as unlikely in the case in *X. vesparum*, due to asynchronous hatching^50^ and the sufficient sperm transfer at the beginning of the copulation to fertilize all of the eggs^31,49^. Furthermore, we found no statistically significant correlation between the number of mating attempts a male made with an already mated female and his reproductive contribution to the offspring. Active or passive displacement of previously deposited sperm by subsequent males, as has been shown in dragonflies^58^, also seem implausible, since Strepsiptera females are inseminated traumatically.

Hrabar et al.^30^ observed that *X. peckii* females protrude the cephalothorax from the host prior to copulation and reinsert it into the host after copulation. This behavior was interpreted as a mechanism to prevent serial mating by multiple males. In our experiments, we also observed significant extrusion (hyperextrusion) of the cephalothorax in *X. vesparum* females. However, we have never observed females retracting their cephalothorax immediately after mating. Interestingly, we even found males successfully mating with females that had not been in the hyperextrusion position at all. Thus, while females may be able to reduce the number of matings by retracting their cephalothorax into the host's body, this does not seem to completely prevent multiple mating and thus polyandry per se. In fact, females of the two species we studied probably mitigate possible negative consequences of traumatic insemination, such as wounding^22,35,37,59,60^, by morphological adaptations (e.g. thickening of the cuticle at the penetration site^22^) rather than by reducing the number of matings. Strepsipteran females may benefit from mating with multiple males. Possible benefits include an increased number of fertilized eggs, an increased genetic diversity of the offspring, and reduced effects of mating with a sterile male^9,11,61,62^. In the light of the present results, we suggest that females either benefit from copulating with multiple males or tolerate polyandry because there is no cost^22,59^.

The fact that the copulation duration of the second mating male of *S. ovinae* is on average shorter than that of the first mating male suggests that males are able to perceive the mating status of a female. Possible cues include the ejaculate of a previous male, as in bed bugs^63^, hemolymph from a previous mating wound, pheromones released by another male on the host's abdomen, or specialized pheromones produced by mated females^20^. Another possible signal perceived by males could be the decrease in sex pheromone released by females after copulation. Tolasch et al.^33^ not only described the chemical structure of the sex pheromone compound (i.e., stylopsal^64^), but also monitored its release from a female. They found that the amount of pheromone secreted by a female decreases significantly immediately after copulation. Perception of mating status with the copulatory organ was ruled out by Peinert et al.^20^*,* because there are no sensilla on the penis. The perception of the female's mating status is only one important aspect for the male to consider. When it comes to strategic ejaculation, as detailed in a meta-analysis by Kelly and Jennions^65^, other factors may also play a role, such as the number of rivals or the mating condition of the female. Wedell and Cook^66^ showed that the small white *Pieris rapae* (Lepidoptera: Pieridae) can strategically determine the amount of sperm to be ejaculated, given that it is a limited resource, depending on the probability of remating and the degree of sperm competition. Comparable data from Strepsiptera are not available, but strategic ejaculation could in principle also play a role in the mating strategies of this taxon. However, as the life span of male Strepsiptera is too short to produce new sperm after ejaculation, unlike Pieridae and other groups, this seems very unlikely.

## Conclusion

Our study showed that, contrary to Kathirithamby et al.^28^, polyandry occurs not only in laboratory experiments with strepsipterans^20^, but also in natural populations. Given the largely uniform morphology and (as far as is known) uniform behavior of Strepsiptera, this tentatively suggests that polyandry may be a ground plan feature of this insect order. Representatives of more families need to be studied for a firm conclusion. However, this hypothesis would be consistent with Kokko and Mappes^67^ who stated that polyandry rather than monandry should serve as the 'null hypothesis'. The results of our experiments with *S. ovinae* are consistent with the mate guarding hypothesis of Peinert et al.^20^ in that the copulation duration of the first mating male negatively affects the fertilization success of a second male. The prolonged copulation of the first male most likely reduces the access of competing males to the female, thereby increasing the proportion of the first male's genetic contribution to the female's offspring (Supplementary Video S2). In contrast, our data do not suggest mate guarding in *X. vesparum.*

## Materials and methods

### Sample collection, rearing, and mating experiments

#### Field samples

To detect polyandry of *S. ovinae* and *X. vesparum* in nature, we collected fertilised females of both species and determined the number of alleles at different polymorphic loci in the offspring of each female. Specifically, to assess the extent of polyandry in *S. ovinae,* HP and KJ collected 50 host bees (*A. vaga*) parasitised by a total of 59 *S. ovinae* females in the Teverner Heide nature reserve (North Rhine-Westphalia, Germany). The bees were collected on a warm day (ca. 16 °C at noon) (February 28, 2022) when male *S. ovinae* were hatching in large numbers and mating with females. Given the short lifespan and flight period of the males, it is reasonable to assume that most (if not all) of the collected females were fertilized by at least one male. To rear primary larvae from the presumably fertilized females collected in the field, we kept the stylopised host bees under stable light in 0.5–l glass jars half- filled with moist sand and sealed with gaze in the laboratory for several weeks until the primary larvae began to hatch. The temperature in a climatic chamber was regulated as follows: 14 °C from 7 a.m. to 10 a.m., 19 °C from 10 a.m. to 5 p.m., 14 °C from 5 p.m. to 6 p.m., and 11 °C from 6 p.m. to 7 a.m. The light was turned on at 7 a.m. and turned off at 6 p.m., resulting in a regular photoperiod of 11 h. The host bees were provided with diluted honey and water ad libitum. Females were visually inspected daily with a tenfold magnifying glass for the appearance of primary larvae, which were easily seen when the larvae appeared at the birth opening. Hatching was accompanied by darkening of the female cephalothorax, caused by the primary larvae inside (Fig. 1A). Females with offspring were removed from the host bee with fine forceps and stored in pure ethanol at − 15 °C. Of the 59 *S. ovinae* females collected, 31 produced primary larvae. The larvae hatched between April 5 and April 20.

The females of *X. vesparum* were collected together with their host, *P. dominula*, on July 7, 2021, in Mettenheim (Rhineland-Palatinate, Germany) by HP and KJ. We collected 29 wasps with a total of 33 *X. vesparum* females, all of which had primary larvae at the birth opening (Fig. 1D). The presence of primary larvae made it unnecessary to keep *X. vesparum* females and their host alive in the laboratory for an extended period of time. Females were removed from their hosts in the laboratory with fine forceps and stored in pure ethanol at − 15 °C. As some hosts contained several females, special care was taken to avoid cross-contamination between the tissues of the females and their primary larvae.

### Laboratory mating experiments

To assess whether the first mating male fertilizes a higher number of offspring than subsequently mating males, we performed paternity tests on parents and offspring of *S. ovinae* and *X. vesparum* in controlled laboratory mating experiments. In the case of *S. ovinae*, the necessary experiments had already been performed by Peinert et al.^20^, whose samples were available to us and suitable for DNA genotyping. The description of their mating experiments is given below. Note that Peinert et al.^20^ provided detailed unpublished information on the parents of all offspring, the mating sequence, and the time and duration of the copulations.

To determine the duration and frequency of copulations, 68 live specimens of *A. vaga*, each parasitized by a single virgin female of *S. ovinae*, were placed separately in glass jars (0.5 l) lined with absorbent paper (to prevent the males from sticking to the excretions of the host bees) at 21 ± 1 °C. A cold light source was used for illumination. A freshly hatched male of *S. ovinae* was placed in each glass jar. After the first copulation, the males were left in the jar for ca. 10 min. The first male was the removed and a second freshly hatched male was added to each of the 58 females. To assess the duration of female attractiveness after copulation, a single newly hatched male was added to 17 *A. vaga* individuals, each parasitized by a single *S. ovinae* female, from 50 min to 3 h 18 min after the first copulation after^20^. In each experiment, the behavior of the male was recorded.

We genotyped the parents and offspring from a total of ten mating trials. The males (fathers) were stored separately in pure ethanol. The bees containing the females (mothers) were kept in a climate chamber under the following controlled conditions until the emergence of the primary larvae: 14 °C from 7 a.m. to 10 a.m., 18 °C from 10 a.m. to 5 p.m., 14 °C from 5 p.m. to 6 p.m., and 10 °C from 6 p.m. to 7 a.m. The light was turned on at 7 a.m. and turned off at 6 p.m., resulting in a regular photoperiod of 11 h. The bees were kept separately in 0.5–l glass jars half filled with moist sand, sealed with gaze, and provided with diluted honey and water ad libitum. After the emergence of the primary larvae, we dissected the bees, removed the *S. ovinae* female(s), and stored them separately with the primary larvae in 100% pure ethanol.

We set up 18 mating experiments with samples of *X. vesparum* similar in design to those performed by Peinert et al.^20^ with *S. ovinae*. However, Peinert et al.^20^ studied freshly hatched males in their experiments, whereas we studied *X. vesparum* males attracted by unmated females in the field. Experiments were conducted in 2018, 2020, and 2021 using samples collected from the sites listed in Supplementary Table S1. In each experiment, the host wasp with an unmated female of *X. vesparum* was fixed in a plastic tube (20 mm long, 7 mm diameter) with a short thread attached to the wasp’s waist, leaving only the metasoma protruding from the tube. To ensure that the strepsipteran male only had access to the protruding wasp metasoma containing the strepsipteran female and to no other regions of the wasp, the end of the tube with the wasp’s head and the space between the wasp’s metasoma and the tube were filled with cotton to ensure that the males did not get stuck in the tube. The tubes were then individually placed in containers (105 mm × 65 mm × 45 mm), in which the copulation experiments were performed, and fixed with plasticine. We started each experiment by releasing a single *X. vesparum* male into a container (Supplementary Video 1), where it remained until it copulated with the female and made no further copulation attempts within 3 min. The male was then removed from the container and fixed in pure ethanol. We then released a second male into the container and repeated the procedure. After the second male had mated, each wasp was placed in individual 0.5–l glass jars sealed with gaze, kept at room temperature (ca. 20 °C), and fed ad libitum with diluted honey and water for up to three months. We initiated artificial hibernation of the host wasps for 12 weeks according to the procedure described by Gibo (1977). Hibernation was terminated by raising the ambient temperature to ca. 20 °C. The wasps and their strepsipteran parasites were checked daily for the appearance of primary larvae. As soon as they appeared, the females were dissected from the host wasps and stored in pure ethanol. In a few cases, the host wasps died before the release of primary larvae from their parasite(s). Nevertheless, we included the offspring in our paternity tests, as these did not depend on fully developed primary larvae. The duration of each copulation and the time between copulations were documented and analysed as described above or as described by Peinert et al.^20^ (Supplementary Tables S2, S3, S4, S5). Due to time and budget constraints, the proportions of offspring produced by the first male and by the second male were calculated from samples of ten of the 18 copulation experiments.

### Tissue dissection and DNA extraction

All specimens were dissected under an Olympus SZ61 Zoom Stereo Microscope (Olympus, Shinyuku, Japan). DNA was extracted from tissues of three legs and of pterothoracic muscles of males, from cephalothoracic muscle tissue of females, and from whole primary larvae. To study the genetic contribution of a given male to the offspring of a female in the laboratory experiments, we extracted the DNA from individual larvae (40 per female and species). The chosen sample size of 40 individuals represented a reasonable trade-off between the time and budget available and the precision of the results obtained (i.e., is the second mating male able to fertilize a major fraction of the large number of eggs of the female; ca. 29,000 in *S. ovinae*, unpublished data H. Pohl, H. Stark). In contrast, to assess the occurrence and the extent of polyandry in fertilized females collected in the field, we extracted DNA of batches of 50 primary larvae (one batch per female, ten batches per species). The DNA was extracted using the QIAamp DNA Micro Kit (Qiagen, Venlo, The Netherlands) according to the manufacturer's protocol.

### Microsatellite marker design

We screened unpublished shotgun genome sequence data of *Stylops ovinae* (courtesy of Leibniz-Institut für die Analyse des Biodiversitätswandels, Museum Koenig, Bonn, Germany) and published shotgun genome sequence data of *Xenos vesparum*^68^ for dinucleotide tandem repeats using the Microsatfinder^69^ software. We then used primer3^70^ to design oligonucleotide primers for PCR amplification of eight identified tandem repeats in each of the two species studied (Supplementary Tables S6 and S7). Since we were only interested in whether alleles specific for one (and only one) of the potential parents had been transmitted to the offspring, and we knew the allelic states of all possible parents, it was not necessary to test for linkage disequilibrium and the occurrence of null alleles.

### PCR

Oligonucleotide primers used for amplification of microsatellite loci by polymerase chain reaction (PCR) are listed in Supplementary Tables S6 and S7. PCRs were performed using the Invitrogen Taq DNA Polymerase Standard PCR Kit (Thermo Fisher Scientific, Waltham, USA), and oligonucleotide primers were produced by Metabion (Munich, Germany). The PCR temperature profile used began with an initial denaturation step of 5 min at 95 °C, followed by 35 cycles of 20 s at 95 °C, 20 s at the annealing temperatures listed in Supplementary Tables S6 and S7, and 30 s at 72 °C. The profile ended with an extension step of 10 min at 72 °C. To reduce the number of PCRs required to assess paternity of offspring, we first genotyped the parents of the samples collected in the laboratory experiments. We then selected a subset of up to four microsatellites to genotype the offspring based on the following criteria: (1) males in a given mating did not share a common allele; (2) no or minimal uncertainty in the size of the microsatellite marker (e.g., due to stutter bands).

### Paternity analysis

We used an ABI 3130xL Genetic Analyzer (Thermo Fisher Scientific, Waltham, USA) at the Department of Forensic Medicine at the University of Freiburg Medical Centre to determine the size of the PCR-amplified microsatellite loci. The samples analysed consisted of 2 µl PCR product, 10 µl Hi-Di™-Formamide (Thermo Fisher Scientific, Waltham, USA), and 0.5 µl of Red 500 DNA Size Standard 500 bp (NimaGen, Nijmegen, The Netherlands). The resulting electropherograms were analysed using the Microsatellite plug-in of the Geneious Prime software version 2021.0.3 (Biomatters, Auckland, New Zealand). The minimum number of fathers in pooled samples of 50 larvae each was inferred from the number of allele states at a given locus in the offspring. For example, five or more detected allele states indicated at least two fathers, since one female and one male can explain a maximum of four alleles in a diploid organism.

### Statistics

Statistical analyses were performed in RStudio version 2021.09.2 (R Core Team, Auckland, New Zealand). We tested whether the distribution of the inferred paternity proportions deviated from a normal distribution using the Shapiro–Wilk test (*Stylops*: W = 0.85401, p-value = 0.06483; *Xenos*: W = 0.85798, p-value = 0.07224). To show whether a particular male contributed more to the offspring of a female than another male, we used a chi-squared goodness of fit test. The influence of the copulation duration on the proportion of offspring was assessed using a generalized linear model (function: glm) in RStudio (R Core Team, Auckland, New Zealand). We used a Poisson distributed model to test whether the total copulation duration of a first copulating male affected the fertilization success (response variable) of a second male. The copulation duration of the second male was included as an additional parameter in the generalized linear model.

### Imaging

All images were processed using Adobe Photoshop version 21.2.1 (Adobe Systems, San Jose, USA). We used Adobe Illustrator version 24.2.1 (Adobe Systems, San Jose, USA) for labeling plates and editing diagrams.

### Supplementary Information

Supplementary Legends.

Supplementary Video 1.

Supplementary Video 2.

Supplementary Tables.

## Data Availability

All data necessary to evaluate the conclusions of the paper are included in the paper and its supplementary information.

## References

[1] Parker GA (1970). Sperm competition and its evolutionary consequences in the insects. Biol. Rev..

[2] Bateman AJ (1948). Intra-sexual selection in *Drosophila*. Heredity.

[3] Darwin, C. *The Descent of Man and Selection in Relation to Sex*. (1871).

[4] André GI, Firman RC, Simmons LW (2020). Baculum shape and paternity success in house mice: Evidence for genital coevolution. Philos. Trans. R. Soc. B Biol. Sci..

[5] Birkhead T, Møller A (1992). Numbers and size of sperm storage tubules and the duration of sperm storage in birds: A comparative study. Biol. J. Linn. Soc..

[6] Firman RC (2020). Of mice and women: Advances in mammalian sperm competition with a focus on the female perspective. Philos. Trans. R. Soc. B Biol. Sci..

[7] Fitzpatrick JL (2020). Sperm competition and fertilization mode in fishes. Philos. Trans. R. Soc. B Biol. Sci..

[8] Taylor ML, Price TA, Wedell N (2014). Polyandry in nature: A global analysis. Trends Ecol. Evol..

[9] Parker GA, Birkhead TR (2013). Polyandry: The history of a revolution. Philos. Trans. R. Soc. B Biol. Sci..

[10] Simmons, L. W. *Sperm Competition and Its Evolutionary Consequences in the Insects* (Princeton University Press, 2001).

[11] Slatyer RA, Mautz BS, Backwell PR, Jennions MD (2012). Estimating genetic benefits of polyandry from experimental studies: A meta-analysis. Biol. Rev..

[12] Parker GA (2006). Sexual conflict over mating and fertilization: An overview. Philos. Trans. R. Soc. B Biol. Sci..

[13] Parker GA, Pizzari T (2010). Sperm competition and ejaculate economics. Biol. Rev..

[14] Pascini TV, Martins GF (2017). The insect spermatheca: An overview. Zoology.

[15] Córdoba-Aguilar A, Uhía E, Rivera AC (2003). Sperm competition in Odonata (Insecta): The evolution of female sperm storage and rivals' sperm displacement. J. Zool..

[16] Uhl G, Nessler SH, Schneider JM (2010). Securing paternity in spiders? A review on occurrence and effects of mating plugs and male genital mutilation. Genetica.

[17] Matzke M (2022). Sperm competition intensity affects sperm precedence patterns in a polyandrous gift-giving spider. Mol. Ecol..

[18] Pohl H, Beutel RG (2005). The phylogeny of Strepsiptera (Hexapoda). Cladistics.

[19] Pohl H, Beutel RG (2008). The evolution of Strepsiptera (Hexapoda). Zoology.

[20] Peinert M (2016). Traumatic insemination and female counter-adaptation in Strepsiptera (Insecta). Sci. Rep..

[21] Jandausch, K., van de Kamp, T., Beutel, R. G., Niehuis, O. & Pohl, H. “Stab, chase me, mate with me, seduce me.” How widespread is traumatic insemination in Strepsiptera? *Biol. J. Linn. Soc.* blad046 (2023).

[22] Jandausch K (2022). Have female twisted-wing parasites (Insecta: Strepsiptera) evolved tolerance traits as response to traumatic penetration?. PeerJ.

[23] Kirkpatrick TW (1937). Studies on the ecology of coffee plantations in East Africa. II. The autecology of *Antestia* spp. (Pentatomidae) with a particular account of a strepsipterous parasite. Trans. Ent. Soc. Lond..

[24] Riek, E. F. *The Insects of Australia* 622–635 (Melbourne Univerity Press, 1970).

[25] Silvestri, F. Studi sugli "Strepsiptera" (Insecta). II. Descrizione, biologia e sviluppo postembrionale dell' *Halictophagus tettigometrae*. *Boll. Lab. Zool. gen. Fac. Agrar. Portici* **32**, 11–48 (1941).

[26] Kinzelbach, R. *Morphologische Befunde an Fächerflüglern und ihre phylogenetische Bedeutung (Insecta: Strepsiptera).* (Zoologica. E. Schweizerbart’sche Verlagsbuchhandlung (Nägele u. Obermiller), 1971).

[27] Kinzelbach R (1978). Fächerflügler (Strepsiptera).

[28] Kathirithamby J (2015). We do not select, nor are we choosy: Reproductive biology of Strepsiptera (Insecta). Biol. J. Linn. Soc..

[29] Dallai R (2004). Fine structure of the Nassonow’s gland in the neotenic endoparasitic of female *Xenos vesparum* (Rossi)(Strepsiptera, Insecta). Tissue Cell.

[30] Hrabar M, Danci A, McCann S, Schaefer PW, Gries G (2014). New findings on life history traits of *Xenos peckii* (Strepsiptera: Xenidae). Can. Entomol..

[31] Grabert, B. *Bau der Geschlechtsorgane und Kopulation beim* Stylops*-Männchen (Insecta, Strepsiptera)* Diploma Thesis thesis, Freie Universität Berlin (1953).

[32] Linsley E, MacSwain J (1957). Observation of the life habits of *Stylops pacifica* Bohart (Coleoptera: Stylopidae). Univ. Calif. Publ. Entomol..

[33] Tolasch T, Kehl S, Dötterl S (2012). First sex pheromone of the order Strepsiptera:(3 R, 5 R, 9 R)-3, 5, 9-Trimethyldodecanal in *Stylops melittae* Kirby, 1802. J. Chem. Ecol..

[34] Brand JN, Harmon LJ, Schaerer L (2022). Frequent origins of traumatic insemination involve convergent shifts in sperm and genital morphology. Evol. Lett..

[35] Lange R, Reinhardt K, Michiels NK, Anthes N (2013). Functions, diversity, and evolution of traumatic mating. Biol. Rev..

[36] Řezáč M (2009). The spider *Harpactea sadistica*: Co-evolution of traumatic insemination and complex female genital morphology in spiders. Proc. R. Soc. B.

[37] Tatarnic NJ, Cassis G, Siva-Jothy MT (2014). Traumatic insemination in terrestrial arthropods. Annu. Rev. Entomol..

[38] Tröger D, Stark H, Beutel RG, Pohl H (2023). The morphology of the free-living females of Strepsiptera (Insecta). J. Morphol..

[39] O’Connor B (1959). The coconut treehopper, *Sexava* spp., and its parasites in the Madang district. Papua N. Guinea Agric. J..

[40] Carayon, J. Traumatic insemination and the paragenital system. *Monograph of Cimicidae (Hemiptera, Heteroptera). College ParkMd: Entomological Society of America*, 81–166 (1966).

[41] Eberhard, W. G. *Sexual Selection and Animal Genitalia*, vol. 244 (Harvard University Press, 1985).

[42] Eberhard, W. *Female Control: Sexual Selection by Cryptic Female Choice*, vol. 69 (Princeton University Press, 1996).

[43] Beani L (2005). Mating of *Xenos vesparum* (Rossi) (Strepsiptera, Insecta) revisited. J. Morphol..

[44] Lindberg, H. Der Parasitismus der auf *Chloriona*-Arten lebenden Strepsiptere *Elenchinus chlorionae* n.sp. sowie die Einwirkung derselben auf ihren Wirt. *Acta Zool. Fenn.* 22, 1–179 (1939).

[45] Parker GA (1990). Sperm competition games: Raffles and roles. Proc. R. Soc. Lond. Ser. B. Biol. Sci..

[46] Lähteenaro M (2023). Phylogenomic species delimitation of the twisted-winged parasite genus *Stylops* (Strepsiptera). Syst. Èntomol..

[47] Benda D, Pohl H, Nakase Y, Beutel RG, Straka J (2022). A generic classification of Xenidae (Strepsiptera) based on morphology of female cephalothorax and male cephalotheca with a preliminary checklist of species. ZooKeys.

[48] Lagoutte R (2013). Total synthesis, proof of absolute configuration, and biosynthetic origin of stylopsal, the first isolated sex pheromone of Strepsiptera. Chem. A Eur. J..

[49] Lauterbach G (1954). Begattung und Larvengeburt bei den Strepsipteren. Z. Parasitenk..

[50] Hughes DP, Kathirithamby J, Beani L (2004). Prevalence of the parasite Strepsiptera in adult *Polistes* wasps: Field collections and literature overview. Ethol. Ecol. Evol..

[51] Pohl, H., Niehuis, O., Gloyna, K., Misof, B. & Beutel, R. G. A new species of *Mengenilla* (Insecta, Strepsiptera) from Tunisia. *ZooKeys.* **79** (2012). 10.3897/zookeys.198.2334 PMC3368257 22707907

[52] Engqvist L, Sauer KP (2003). Determinants of sperm transfer in the scorpionfly *Panorpa cognata*: Male variation, female condition and copulation duration. J. Evol. Biol..

[53] Albo MJ, Bilde T, Uhl G (2013). Sperm storage mediated by cryptic female choice for nuptial gifts. Proc. R. Soc. B.

[54] Martin O, Hosken D (2002). Strategic ejaculation in the common dung fly *Sepsis cynipsea*. Anim. Behav..

[55] Carlberg U (1987). Mate choice, sperm competition and storage of sperm in
* Baculum* sp. l (Insecta: Phasmida). Zool. Anz..

[56] Carlberg U (1987). Reproduction behavior of *Extatosoma tiaratum* (MacLeay) (Insecta: Phasmida). Zool. Anz..

[57] Müller J, Eggert A-K (1989). Paternity assurance by “helpful” males: Adaptations to sperm competition in burying beetles. Behav. Ecol. Sociobiol..

[58] Waage JK (1979). Dual function of the damselfly penis: Sperm removal and transfer. Science.

[59] Michels J, Gorb SN, Reinhardt K (2015). Reduction of female copulatory damage by resilin represents evidence for tolerance in sexual conflict. J. R. Soc. Interface.

[60] Reinhardt K, Anthes N, Lange R (2015). Copulatory wounding and traumatic insemination. Cold Spring Harb. Perspect. Biol..

[61] Arnqvist G, Nilsson T (2000). The evolution of polyandry: Multiple mating and female fitness in insects. Anim. Behav..

[62] Tregenza T, Wedell N (1998). Benefits of multiple mates in the cricket *Gryllus bimaculatus*. Evolution.

[63] Siva-Jothy MT, Stutt AD (2003). A matter of taste: Direct detection of female mating status in the bedbug. Proc. R. Soc. B.

[64] Cvačka J (2012). Stylopsal: The first identified female-produced sex pheromone of Strepsiptera. J. Chem. Ecol..

[65] Kelly CD, Jennions MD (2011). Sexual selection and sperm quantity: Meta-analyses of strategic ejaculation. Biol. Rev..

[66] Wedell N, Cook P (1999). Strategic sperm allocation in the small white butterfly *Pieris rapae* (Lepidoptera: Pieridae). Funct. Ecol..

[67] Kokko H, Mappes J (2013). Multiple mating by females is a natural outcome of a null model of mate encounters. Entomol. Exp. Appl..

[68] Mahajan S, Bachtrog D (2015). Partial dosage compensation in Strepsiptera, a sister group of beetles. Genome Biol. Evol..

[69] Thurston, M. & Field, D. Msatfinder: Detection and characterisation of microsatellites. Distributed by the authors at http://www.genomics.ceh.ac.uk/msatfinder/. CEH Oxford, Mansfield Road, Oxford OX1 3SR (2005).

[70] Rozen, S. & Skaletsky, H. *Bioinformatics Methods and Protocols*, vol. 132 365–386 (Humana Press, 2000).

