# Supplementary material for: Polyandry and sperm competition in two traumatically inseminating species of Strepsiptera (Insecta)
Source: Sci Rep. 2024 May 7;14:10447. doi: 10.1038/s41598-024-61109-z (PMC11076583; doi:10.1038/s41598-024-61109-z)
Supplement: Supplementary file 1 — Supplementary Legends. [file 41598_2024_61109_MOESM1_ESM.pdf]

Supplementary Video 1. Copulation of *Xenos vesparum* under laboratory conditions.

Supplementary Video 2. Mating of *Stylops ovinae*. Three males competing for one female extruding from the metasoma of its host bee *Andrena vaga.* ©Katrin Kunkel
