# Supplementary material for: Polyandry and sperm competition in two traumatically inseminating species of Strepsiptera (Insecta)
Source: Sci Rep. 2024 May 7;14:10447. doi: 10.1038/s41598-024-61109-z (PMC11076583; doi:10.1038/s41598-024-61109-z)
Supplement: Supplementary file 4 — Supplementary Tables. [file 41598_2024_61109_MOESM4_ESM.pdf]

Supplementary Information

Polyandry and sperm competition in two traumatically inseminating species of Strepsiptera (Insecta)

Table S1: Geographic origin of males and females of *Xenos vesparum* studied in laboratory mating experiments and dates we conducted the mating experiments and hibernation.

Year	Geographic origin of males	Geographic origin of females	Type of vessel used	Number of mating experiments
**2018**	Mettenheim, Rhineland-Palatinate, Germany; hatched from host; 07/2018	breeding ex. *Polistes nympha* (D, Thuringia, Rothenstein, June 2018) infected with primary larva, D, Baden-Württemberg, Kaiserstuhl, Oberbergen, July 2018, leg. H. Pohl	Glass vessel (0.5 l)	4
**2020**	Jena, Thuringia, Germany; attracted with female; July 2020	Mettenheim, Rhineland-Palatinate, Germany; July 2020	Plastic box (105 mm x 65 mm x 45 mm)	5
**2021**	Jena, Thuringia, Germany; attracted with female; July 2021	Mettenheim, Rhineland-Palatinate, Germany; July 2021	Plastic box (105 mm x 65 mm x 45 mm)	9

Table S2. Mating experiments of *Stylops ovinae* under laboratory conditons.

ID from Peinert 2016	Lab ID	Sex of host	Female *Stylops*	Male *Stylops*	Copulation duration								Copulation (day)	Specimen fixed
					male 1			male 2						
16	So01	female	1	0	12 min 46 s			5 min 8 s					28.01.2014	29.03.2014
17	So02	female	1	0	24 s	1 min 23 s	1 min 1 s	46 s	13 s	9 s			30.01.2014	29.03.2014
18	So03	female	1	0	8 min 40 s			1 min 56 s	8 s				30.01.2014	17.03.2014
22	So04	female	1	1	1 min 17 s			1 m 34 s	15 s	6 s	4 min 9 s	15 s	31.01.2014	26.03.2014
25	So05	female	1	0	34 s			13 m 33 s	50 s				01.02.2014	29.03.2014
29	So06	female	1	0	27 s			15 s					20.02.2014	29.03.2014
30	So07	female	1	0	2 min 13 s			6 min 28 s					20.02.2014	11.04.2014
33	So08	female	1	0	16 min 2 s			6 min 9 s	11 s				21.02.2014	11.04.2014
39	So09	female	1	0	33 s	39 s	11 s	1 min 15 s	15 s	23 s			23.02.2014	10.04.2014
52	So10	female	1	0	12 min 49 s			13 s					04.03.2014	11.04.2014

Table S3. Mating experiments of *Xenos vesparum* under laboratory conditons.

ID	Lab ID	Sex of host	Female *Xenos*	Copulation duration											Copula (day)	Begin of hibernation	End of hibernation	Specimen fixed
				male1				male2						male 3				
Pol_01		female	1												10.07.2018	no hiberna-tion	–	not fixed
Pol_02		female	1												11.07.2018	no hiberna-tion	–	not fixed
Pol_03	XL1	female	1	23 s				5 s	13 s						11.07.2018	06.11.2018	died during hibernation	15.11.2018
Pol_04		female	1	16 s				3 s	7 s						12.07.2018	06.11.2018	29.01.2019	18.03.2019
Pol_05		female	1	34 s				8 s	10 s	2 s					13.07.2018	06.11.2018	29.01.2019	17.03.2019
Pol_06		female	1	30 s				9 s	15 s	15 s	1 s				16.07.2018	06.11.2018	29.01.2019	31.01.2019
Pol_07		female	1	30 s				5 s						5 s	19.07.2020	no hiberna-tion	–	19.07.2020
Pol_08		female	1	5 s	20 s	29 s		5 s	2 s	9 s	10 s				24.07.2020	07.10.2020	17.12.2020	03.01.2021
Pol_09	XL2	female	1	9 s	5 s	6 s		4 s	3 s						24.07.2020	died before hibernation	–	04.08.2020
Pol_10	XL3	female	1	3 s	1 s	2 s		2 s	2 s	3 s					27.07.2020	07.10.2020	17.12.2020	15.02.2021
Pol_11		female	1	3 s				5 s	2 s	several short attempts					05.08.2020	07.10.2020	died during hibernation	12.10.2020
Pol_12	XL4	female	1	4 s	3 s			7 s	2 s						30.07.2021	13.10.2021	died during hibernation	27.12.2021

Table S3 (cont.). Mating experiments of *Xenos vesparum* under laboratory conditons.

ID	Lab ID	Sex of host	female *Xenos*	Copulation duration											Copula (Day)	Begin of hibernation	End of hi-bernation	Specimen fixed
				male1				male2						male 3				
Pol_13	XL5	female	1	5 s				33 s							30.07.2021	13.10.2021	06.01.2022	09.02.2022
Pol_14	XL6	female	1	4 s	2 s	2 s	3 s	15 s							30.07.2021	13.10.2021	06.01.2022	09.02.2021
Pol_15	XL7	female	1	6 s	2 s			4 s	2 s						30.07.2021	13.10.2021	06.01.2022	07.02.2022
Pol_16	XL8	female	1	5 s	2 s			1 s							03.08.2021	13.10.2021	died during hibernation	04.01.2022
Pol_17	XL9	female	1	4 s				6 s	3 s	3 s	2 s	2 s	2 s		05.08.2021	13.10.2021	died during hibernation	04.11.2021
Pol_18		female	1	14 s	5 s	3 s		15 s							06.08.2021	died before hibernation	–	21.09.2021
Pol_19	XL10	female	1	3 s	5 s			4 s	6 s	5 s	3 s				06.08.2021	13.10.2021	06.01.2022	11.02.2021
Pol_20		male	1	3 s	4 s	3 s		2 s	3 s	1 s					09.08.2021	died before hibernation	–	23.09.2021

Table S4: Duration and frequency of copulation in *X. vesparum*[1].

		Virgin females (n = 18)			Mated females (n =18)		
**Copula**		First n=18	Second n=10	Third n=6	First n=18	Second n=13	Third n=7
**Duration of copula (s)**	Average	11.2	5.9	7.5	7.4	5.4	5.4
	Min.	3	1	2	1	2	1
	Max	34	20	29	33	15	15
	median	5	3.5	3	5	3	3

[1] We recorded the duration and frequency of copulations in *Xenos vesparum* using a similar approach as Peinert et al. (2016) used when studying *Stylops ovinae*. In total, we observed 79 copulations and found the copulation duration to not be normally distribution (Shapiro-Wilk test: W = 0.87311, p-value = 0.0007). In a single instance, a male copulated six times for a few seconds with a female that had previously copulated with another male. We observed no significant difference between the copulation duration of the first copulating male and the copulation duration of the second male (Mann-Whitney U test: W = 185.5, p-value = 0.4634), and no significant difference between the first copulation duration and subsequent copulation duration of a single male. This applies to copulations with virgin females (V = 39.5, p-value = 0.2393) and to previously mated females (V = 36.5, p-value = 0.8749).

Table S5. Documented copulation attempts, copulation duration of the first and of the second male, and time between the onset of the first male's copulation and the onset of the second male's copulation with a given female.

ID	Time between inseminations	Total copulation time M1	Copulation attempts M1	Total copulation time M2	Copulation attempts M2
So01	23 min 19 s	12 min 46 s	1	5 min 8 s	1
So02	13 min 21 s	2 min 48 s	3	1 min 8 s	3
So03	19 min 13 s	8 min 40 s	1	2 min 4 s	2
So04	11 min 56 s	1 min 23 s	3	1 min 53 s	3
So05	11 min 50 s	1 min 17 s	1	6 min 19 s	5
So06	11 min 7 s	0 min 34 s	1	14 min 23 s	2
So07	11 min	0 min 27 s	1	0 min 15 s	1
So08	12 min 46 s	2 min 13 s	1	6 min 28 s	1
So09	26 min 35 s	16 min 2 s	1	6 min 20 s	2
So10	90 min 22 s	12 min 49 s	1	0 min 13 s	1
Xv01	20 min 45 s	23 s	1	18 s	2
Xv02	8 min 34 s	11 s	3	6 s	2
Xv03	2 min 29 s	6 s	3	7 s	3
Xv04	1 min 15 s	7 s	2	9 s	2
Xv05	2 min 25 s	5 s	1	33 s	1
Xv06	3 min 47 s	11 s	4	15 s	1
Xv07	6 min 53 s	8 s	2	6 s	2
Xv08	7 min 33 s	7 s	2	1 s	1
Xv09	4 min 42 s	4 s	1	18 s	4
Xv10	4 min 28 s	8 s	2	18 s	4

Table S6. Characterisation of polymorphic microsatellite markers used in paternity tests on *Stylops ovinae* (So). Abbreviations: bp = base pair; MS, ms=microsatellite;F=forwardoligonucleotideprimer;R=reverseoligonucleotideprimer;HEX=Hexachloro-fluorescein;6-FAM=6-Carboxyfluorescein; TM = melting temperature.

Species	MS	Dinucleotide repeate	Primer	Primer nucleotide sequence 5‘–>3‘	Allele range	5’– modification	TM (°C)	Annealing temperature (°C)
S. oviane	So_A	AC	F	GTGCTATCATTTAATGTTATCGC	147–153	HEX	54.4	51.4
			R	ATAAGAATTGAGCCACGCCC		—	58.3	
	So_B	CA	F	GAGCAAAACTAACTCCATACT	227–247	6-FAM	58.3	50.3
			R	TTTTGCCGGTTTGAAGGAAA		—	56.7	
	So_C	CA	F	ACCTATCCCCATGCATGCTT	186–198	6-FAM	59.1	55.3
			R	CTGTCTGTGTACTGCCATGG		—	58.3	
	So_D	CT	F	CGAAAGCAGACATAACGATTTCT	142–158	6-FAM	57.5	54.3
			R	TCGAGGCTTCCAATAAGACATT		—	57.5	
	So_E	GA	F	TGTTGTTGTCTTTTGTTTCTACA	101–111	6-FAM	55.2	52.1
			R	TCGAGCACTATGATTTGATGT			55.1	
	So_F	GT	F	ATTTGGACCATTTCTTTCCT	220–228	HEX	52.6	49.6
			R	ACGGTTCATTTGATATCGAA		—	52.7	
	So_G	CA	F	TGTTGTAATCGAAAAGTCTGCAT	179–191	HEX	57.1	54.1
			R	ATCAGCAAGTCTATAACCATGGT		—	57.4	
	So_H	AC	F	TCTTATTATTAGACACCCACTG	105 –113	HEX	52.7	49.7
			R	CGGCGGAACATTTTGAGAAA		—	57.3	

Table S7. Characterisation of polymorphic microsatellite markers used in paternity tests on *Xenos vesparum* (Xv). Abbreviations: bp = base pair; MS, ms=microsatellite; F=forward oligonucleotide primer; R=reverse oligonucleotide primer; HEX=Hexachloro-fluorescein; 6-FAM=6-Carboxyfluorescein; TM = melting temperature.

Species	MS	Dinucleotide repeate	Primer	Primer nucleotide sequence 5'–>3'	Allele range	5'– modification	TM (°C)	Annealing temperature (°C)
X. vesparum	Xv_A	GA	F	TCGAAGTTGTGCAAAGAGTA	169–177	6-FAM	55.0	47.1
			R	GGATTATCTGGAATGCAAT		—	50.1	
	Xv_B	AG	F	GTCGATAGCATATCAAAAGCC	222–238	6-FAM	54.9	51.9
			R	CTCCGACACTCAGATTTTGA		—	55.1	
	Xv_C	AC	F	GAAGAATAGAAAAGTCMGGAAAA	190–200	6-FAM	52.7	49.7
			R	GTCACTTGGTACGGTACAGT		—	56.9	
	Xv_D	TG	F	TTTTCTTCATTTCGGTCGTA	132–148	HEX	52.9	49.9
			R	ACACATCGGGCGAGAAAA		—	56.9	
	Xv_E	TC	F	CGCTATACTGTCTATTCTCCGC	174–184	HEX	58.3	55.3
			R	CGCGAGCCGTTTTGTATTTC		—	58.5	
	Xv_F	CA	F	ACACCGAGTTAAGAATTATCCA	228–236	HEX	54.9	51.9
			R	GTCGAAGTGGCCATAAGTAA		—	55.2	
	Xv_G	TG	F	GTTGTGTATTGTATGCGTTCGG	131–133	HEX	58.3	55.3
			R	CCCTAGTGATTGAAGCGTGG		—	58.3	
	Xv_H	TG	F	TGTTTAATGAGAGTGTACCTGT	247–253	6-FAM	54.7	51.0
			R	TTTCGGTATCTTTGACTGGA			54.0	

Table S8. Allele states for tested larva and parents (green) in *Stylops ovinae* (So_01-10).

So_01	Locus A.1	Locus A.2	Locus B.1	Locus B.2	Locus C.1	Locus C.2	Locus G.1	Locus G.2
**Female**	153	159	228	242	192	194	181	185
**Male 1**	151	151	242	242	186	186	185	187
**Male 2**	147	151	228	246	194	198	178	183
**Larva 01**	151	153	242	242	186	192	185	185
**Larva 02**	151	153	228	242	186	192	185	185
**Larva 03**	151	159	242	242	186	192	181	185
**Larva 04**	151	153	242	242	186	192	181	187
**Larva 05**	151	153	228	242	186	194	185	187
**Larva 06**	151	159	228	242	186	192	185	185
**Larva 07**	151	153	228	242	186	194	181	185
**Larva 08**	151	159	242	242	186	194	185	187
**Larva 09**	151	153	228	242	186	194	181	185
**Larva 10**	151	153	228	242	186	192	181	187
**Larva 11**	151	153	242	242	186	192	181	187
**Larva 12**	151	159	242	242	186	194	181	187
**Larva 13**	151	153	242	242	186	192	N.A.	N.A.
**Larva 14**	151	159	228	242	186	194	181	185
**Larva 15**	151	153	242	242	186	194	181	185
**Larva 16**	151	159	228	242	186	194	185	185
**Larva 17**	151	153	242	242	186	192	185	185
**Larva 18**	151	159	228	242	186	194	181	187
**Larva 19**	151	153	228	242	186	192	181	185
**Larva 20**	147	153	228	228	192	194	178	185
**Larva 21**	151	159	242	242	186	192	181	185
**Larva 22**	147	151	228	246	192	194	178	185
**Larva 23**	151	159	228	242	186	194	181	185
**Larva 24**	151	159	228	242	186	192	181	185
**Larva 25**	151	153	N.A.	N.A.	186	194	181	187
**Larva 26**	151	159	228	246	194	194	181	183
**Larva 27**	147	159	N.A.	N.A.	194	194	178	185
**Larva 28**	151	159	242	246	192	194	178	185
**Larva 29**	151	159	228	242	186	192	185	187
**Larva 30**	151	153	228	242	186	192	185	187
**Larva 31**	151	159	242	242	186	192	185	187
**Larva 32**	151	153	228	242	186	194	185	187
**Larva 33**	151	153	242	242	186	194	185	185
**Larva 34**	151	159	242	242	186	192	185	185
**Larva 35**	151	153	228	242	186	192	185	185
**Larva 36**	151	159	228	242	186	194	185	187
**Larva 37**	151	153	242	242	186	192	185	187
**Larva 38**	151	153	228	242	186	194	181	187
**Larva 39**	151	153	228	242	186	194	181	187
**Larva 40**	151	153	N.A.	N.A.	186	192	185	185

So_02	Locus A.1	Locus A.2	Locus B.1	Locus B.2	Locus C.1	Locus C.2	Locus D.1	Locus D.2
**Female**	149	151	228	230	187	187	152	156
**Male 1**	151	159	242	250	187	187	156	164
**Male 2**	145	151	236	250	187	193	140	156
**Larva 01**	149	159	230	242	187	187	152	164
**Larva 02**	149	151	230	242	187	187	152	156
**Larva 03**	149	151	230	242	187	187	156	164
**Larva 04**	151	151	230	250	N.A.	N.A.	156	164
**Larva 05**	151	151	228	242	N.A.	N.A.	152	164
**Larva 06**	151	159	228	242	N.A.	N.A.	156	156
**Larva 07**	149	151	228	250	187	187	152	156
**Larva 08**	151	159	230	242	N.A.	N.A.	156	164
**Larva 09**	149	151	228	236	187	193	156	156
**Larva 10**	151	159	230	242	N.A.	N.A.	152	164
**Larva 11**	149	151	228	250	N.A.	N.A.	156	164
**Larva 12**	149	151	N.A.	N.A.	187	193	152	156
**Larva 13**	151	159	228	250	N.A.	N.A.	152	164
**Larva 14**	149	151	230	236	187	187	140	156
**Larva 15**	151	159	228	242	N.A.	N.A.	156	164
**Larva 16**	149	159	228	250	N.A.	N.A.	156	164
**Larva 17**	149	151	230	242	N.A.	N.A.	156	156
**Larva 18**	149	159	230	242	N.A.	N.A.	152	164
**Larva 19**	151	159	230	250	N.A.	N.A.	156	156
**Larva 20**	151	151	230	242	N.A.	N.A.	156	156
**Larva 21**	149	151	230	250	N.A.	N.A.	152	164
**Larva 22**	151	151	228	242	N.A.	N.A.	152	164
**Larva 23**	151	159	230	242	N.A.	N.A.	152	164
**Larva 24**	151	151	228	250	187	187	156	156
**Larva 25**	151	151	230	242	N.A.	N.A.	156	164
**Larva 26**	149	159	230	242	N.A.	N.A.	156	164
**Larva 27**	151	151	228	242	N.A.	N.A.	156	156
**Larva 28**	149	159	230	250	N.A.	N.A.	152	156
**Larva 29**	151	151	228	242	N.A.	N.A.	152	156
**Larva 30**	151	151	228	250	N.A.	N.A.	156	164
**Larva 31**	151	151	230	250	187	193	152	156
**Larva 32**	145	151	230	236	187	187	140	152
**Larva 33**	149	159	230	242	N.A.	N.A.	152	156
**Larva 34**	149	151	N.A.	N.A.	N.A.	N.A.	156	164
**Larva 35**	151	151	228	242	N.A.	N.A.	152	156
**Larva 36**	149	151	230	242	N.A.	N.A.	152	164
**Larva 37**	151	151	228	242	N.A.	N.A.	156	156
**Larva 38**	151	151	228	250	N.A.	N.A.	156	156
**Larva 39**	149	151	228	250	187	187	152	156
**Larva 40**	149	159	230	242	N.A.	N.A.	152	164

So_03	Locus B.1	Locus B.2	Locus D.1	Locus D.2	Locus E.1	Locus E.2
**Female**	250	250	140	164	99	99
**Male 1**	228	242	154	156	99	105
**Male 2**	246	250	140	156	105	109
**Larva 01**	250	250	140	140	99	105
**Larva 02**	228	250	154	164	99	105
**Larva 03**	250	250	140	164	99	105
**Larva 04**	242	250	156	164	99	105
**Larva 05**	250	250	140	164	99	109
**Larva 06**	250	250	140	164	99	105
**Larva 07**	242	250	154	164	99	99
**Larva 08**	246	250	140	164	99	109
**Larva 09**	250	250	140	164	99	109
**Larva 10**	246	250	156	164	99	109
**Larva 11**	242	250	156	164	99	105
**Larva 12**	228	250	156	164	99	99
**Larva 13**	228	250	154	164	99	105
**Larva 14**	246	250	140	164	99	109
**Larva 15**	246	250	156	164	99	105
**Larva 16**	246	250	156	164	99	105
**Larva 17**	250	250	140	164	99	105
**Larva 18**	246	250	140	164	99	109
**Larva 19**	250	250	140	164	99	109
**Larva 20**	242	250	154	164	99	105
**Larva 21**	246	250	140	164	99	105
**Larva 22**	228	250	156	164	99	105
**Larva 23**	246	250	140	164	99	105
**Larva 24**	246	250	156	164	99	105
**Larva 25**	242	250	156	164	99	99
**Larva 26**	228	250	156	164	99	99
**Larva 27**	250	250	140	164	99	105
**Larva 28**	246	250	156	164	99	109
**Larva 29**	228	250	154	164	99	105
**Larva 30**	228	250	154	164	99	105
**Larva 31**	228	250	154	164	99	99
**Larva 32**	242	250	156	164	99	105
**Larva 33**	246	250	156	164	99	109
**Larva 34**	250	250	140	164	99	105
**Larva 35**	242	250	154	164	99	105
**Larva 36**	228	250	154	164	99	99
**Larva 37**	250	250	140	164	99	109
**Larva 38**	228	250	156	164	99	105
**Larva 39**	228	250	154	164	99	99
**Larva 40**	250	250	156	164	99	109

So_04	Locus B.1	Locus B.2	Locus D.1	Locus D.2	Locus G.1	Locus G.2
**Female**	228	242	150	154	179	185
**Male 1**	230	246	150	156	179	191
**Male 2**	214	214	150	154	191	193
**Larva 01**	214	228	154	154	185	191
**Larva 02**	214	242	150	154	N.A.	N.A.
**Larva 03**	214	228	154	154	185	191
**Larva 04**	214	228	154	154	179	193
**Larva 05**	214	228	150	154	185	193
**Larva 06**	242	246	150	150	179	179
**Larva 07**	214	228	154	154	185	191
**Larva 08**	214	228	150	154	179	193
**Larva 09**	228	246	154	156	185	191
**Larva 10**	242	246	154	156	185	191
**Larva 11**	242	246	150	156	179	185
**Larva 12**	214	242	154	154	179	191
**Larva 13**	214	242	150	154	179	191
**Larva 14**	214	228	154	154	179	191
**Larva 15**	214	228	150	150	185	191
**Larva 16**	230	242	154	156	179	191
**Larva 17**	230	242	150	150	185	191
**Larva 18**	230	242	154	156	179	191
**Larva 19**	242	246	154	156	179	179
**Larva 20**	228	246	154	156	179	191
**Larva 21**	214	242	150	150	185	193
**Larva 22**	214	242	150	154	185	193
**Larva 23**	228	246	150	154	179	185
**Larva 24**	228	230	150	156	185	191
**Larva 25**	228	246	150	154	179	191
**Larva 26**	214	228	150	150	185	193
**Larva 27**	214	242	150	154	179	191
**Larva 28**	214	242	150	150	185	191
**Larva 29**	242	246	154	156	179	191
**Larva 30**	228	246	150	150	179	185
**Larva 31**	214	242	150	154	185	191
**Larva 32**	214	242	150	154	179	193
**Larva 33**	214	228	150	154	185	193
**Larva 34**	214	242	150	150	179	191
**Larva 35**	214	228	150	154	179	193
**Larva 36**	214	228	154	154	179	193
**Larva 37**	214	242	154	154	179	193
**Larva 38**	214	228	150	154	179	191
**Larva 39**	228	246	150	154	179	185
**Larva 40**	228	230	150	154	179	187

So_05	Locus A.1	Locus A.2	Locus C.1	Locus C.2	Locus G.1	Locus G.2
**Female**	151	159	193	195	185	185
**Male 1**	149	159	187	195	185	185
**Male 2**	147	151	195	199	179	187
**Larva 01**	151	159	193	195	185	185
**Larva 02**	149	151	187	195	185	185
**Larva 03**	151	151	193	195	179	185
**Larva 04**	149	159	193	195	185	185
**Larva 05**	151	159	193	195	179	185
**Larva 06**	159	159	193	195	185	185
**Larva 07**	149	159	193	195	185	185
**Larva 08**	149	151	193	195	185	185
**Larva 09**	149	159	195	195	185	185
**Larva 10**	149	151	187	195	185	185
**Larva 11**	159	159	195	195	185	185
**Larva 12**	151	159	187	193	185	185
**Larva 13**	149	151	193	195	N.A.	N.A.
**Larva 14**	147	151	195	195	179	185
**Larva 15**	151	159	187	195	185	185
**Larva 16**	151	151	193	199	185	187
**Larva 17**	149	159	195	195	185	185
**Larva 18**	151	159	193	195	185	185
**Larva 19**	149	151	187	193	185	185
**Larva 20**	149	159	187	195	185	185
**Larva 21**	151	159	187	195	185	185
**Larva 22**	149	159	187	195	185	185
**Larva 23**	159	159	187	195	185	185
**Larva 24**	149	159	187	193	185	185
**Larva 25**	149	151	187	193	185	185
**Larva 26**	151	159	195	199	185	187
**Larva 27**	149	151	187	195	185	185
**Larva 28**	159	159	187	195	185	185
**Larva 29**	151	159	193	195	185	187
**Larva 30**	151	159	187	193	185	185
**Larva 31**	149	151	187	195	185	185
**Larva 32**	147	159	195	195	185	187
**Larva 33**	159	159	195	195	185	185
**Larva 34**	151	159	187	195	185	185
**Larva 35**	159	159	193	195	185	185
**Larva 36**	147	159	195	195	185	187
**Larva 37**	151	159	193	195	185	185
**Larva 38**	147	151	195	195	185	187
**Larva 39**	151	159	195	195	179	185
**Larva 40**	149	151	187	193	185	185

So_06	Locus A.1	Locus A.2	Locus B.1	Locus B.2
Female	147	147	230	242
Male 1	151	151	242	250
Male 2	147	159	214	242
Larva 01	147	159	214	242
Larva 02	147	151	N.A.	N.A.
Larva 03	147	151	242	242
Larva 04	147	159	230	242
Larva 05	147	147	214	230
Larva 06	147	159	214	242
Larva 07	147	159	214	230
Larva 08	147	151	242	242
Larva 09	147	147	214	230
Larva 10	147	151	230	242
Larva 11	147	147	214	230
Larva 12	147	159	214	230
Larva 13	147	159	242	242
Larva 14	147	151	230	242
Larva 15	147	151	230	242
Larva 16	147	151	230	242
Larva 17	147	151	242	242
Larva 18	147	147	214	230
Larva 19	147	147	214	230
Larva 20	147	151	230	242
Larva 21	147	159	214	230
Larva 22	147	151	242	242
Larva 23	147	159	230	242
Larva 24	147	159	214	242
Larva 25	147	147	214	242
Larva 26	147	151	242	242
Larva 27	147	159	214	242
Larva 28	147	151	242	242
Larva 29	147	147	214	230
Larva 30	147	151	230	242
Larva 31	147	159	214	242
Larva 32	147	159	214	242
Larva 33	147	151	230	242
Larva 34	147	147	214	230
Larva 35	147	151	242	242
Larva 36	147	159	214	230
Larva 37	147	147	214	230
Larva 38	147	151	242	242
Larva 39	147	147	214	230
Larva 40	147	147	214	230

So_07	Locus A.1	Locus A.2	Locus D.1	Locus D.2	Locus G.1	Locus G.2
**Female**	147	151	140	164	183	191
**Male 1**	151	151	150	156	183	185
**Male 2**	149	153	156	158	179	185
**Larva 01**	N.A.	N.A.	156	164	179	191
**Larva 02**	147	153	140	158	179	183
**Larva 03**	147	151	140	156	185	191
**Larva 04**	151	151	140	150	183	191
**Larva 05**	147	151	150	164	185	191
**Larva 06**	147	151	140	156	183	183
**Larva 07**	147	149	158	164	183	185
**Larva 08**	147	151	140	156	183	183
**Larva 09**	147	151	140	156	N.A.	N.A.
**Larva 10**	149	151	158	164	179	191
**Larva 11**	151	151	140	156	183	185
**Larva 12**	149	151	158	164	183	185
**Larva 13**	N.A.	N.A.	140	158	179	191
**Larva 14**	149	151	156	164	179	183
**Larva 15**	151	151	150	164	183	183
**Larva 16**	147	151	150	164	183	183
**Larva 17**	151	151	140	156	183	185
**Larva 18**	151	151	140	150	183	183
**Larva 19**	151	151	140	156	183	185
**Larva 20**	147	151	156	164	183	183
**Larva 21**	147	151	140	156	183	183
**Larva 22**	147	151	156	164	183	183
**Larva 23**	147	151	140	156	185	191
**Larva 24**	147	153	156	164	179	183
**Larva 25**	151	151	140	156	N.A.	N.A.
**Larva 26**	151	151	156	164	183	185
**Larva 27**	147	151	140	150	185	191
**Larva 28**	151	151	140	156	183	183
**Larva 29**	147	151	150	164	N.A.	N.A.
**Larva 30**	151	151	140	156	N.A.	N.A.
**Larva 31**	147	151	150	164	183	191
**Larva 32**	151	153	158	164	179	183
**Larva 33**	151	151	156	164	185	191
**Larva 34**	151	151	156	164	185	191
**Larva 35**	147	149	140	158	179	191
**Larva 36**	N.A.	N.A.	N.A.	N.A.	N.A.	N.A.
**Larva 37**	151	151	140	150	183	183
**Larva 38**	151	151	N.A.	N.A.	183	183
**Larva 39**	151	151	150	164	185	191
**Larva 40**	147	151	150	164	183	191
**Larva 41**	147	151	N.A.	N.A.	N.A.	N.A.

So_08	Locus A.1	Locus A.2	Locus B.1	Locus B.2
**Female**	151	153	214	228
**Male 1**	151	159	244	246
**Male 2**	149	149	230	250
**Larva 01**	151	153	214	246
**Larva 02**	151	153	214	244
**Larva 03**	151	153	228	244
**Larva 04**	153	159	214	246
**Larva 05**	149	153	228	250
**Larva 06**	151	159	214	244
**Larva 07**	153	159	228	246
**Larva 08**	153	159	214	246
**Larva 09**	149	151	228	250
**Larva 10**	151	151	228	246
**Larva 11**	151	151	214	244
**Larva 12**	151	153	228	246
**Larva 13**	153	159	214	246
**Larva 14**	151	153	228	244
**Larva 15**	149	153	214	230
**Larva 16**	151	159	214	246
**Larva 17**	151	159	214	244
**Larva 18**	151	159	228	244
**Larva 19**	149	153	228	250
**Larva 20**	151	151	228	244
**Larva 21**	151	153	214	246
**Larva 22**	151	153	214	246
**Larva 23**	151	153	228	246
**Larva 24**	153	159	228	246
**Larva 25**	149	153	214	230
**Larva 26**	153	159	228	246
**Larva 27**	153	159	214	244
**Larva 28**	151	159	214	244
**Larva 29**	149	153	228	250
**Larva 30**	151	151	214	246
**Larva 31**	151	159	214	244
**Larva 32**	151	153	228	246
**Larva 33**	151	151	228	246
**Larva 34**	151	159	214	244
**Larva 35**	153	159	214	246
**Larva 36**	151	151	228	246
**Larva 37**	151	159	214	244
**Larva 38**	151	151	214	244
**Larva 39**	151	153	214	244
**Larva 40**	153	159	214	246

So_09	Locus B.1	Locus B.2	Locus C.1	Locus C.2	Locus D.1	Locus D.2	Locus G.1	Locus G.2
**Female**	242	250	187	193	140	150	185	187
**Male 1**	214	228	187	187	154	156	178	187
**Male 2**	242	246	197	199	140	156	183	185
**Larva 01**	228	242	187	187	140	156	185	187
**Larva 02**	228	242	187	193	140	154	178	187
**Larva 03**	228	242	187	187	140	154	187	187
**Larva 04**	228	250	187	187	150	156	185	187
**Larva 05**	228	250	187	193	140	156	178	185
**Larva 06**	228	250	N.A.	N.A.	150	154	178	187
**Larva 07**	214	250	187	193	140	156	178	185
**Larva 08**	214	250	187	193	150	154	178	187
**Larva 09**	228	250	187	187	N.A.	N.A.	178	185
**Larva 10**	214	250	187	187	140	156	178	187
**Larva 11**	228	250	187	193	150	154	185	187
**Larva 12**	228	242	187	193	N.A.	N.A.	N.A.	N.A.
**Larva 13**	214	242	187	187	140	156	185	187
**Larva 14**	214	250	187	187	140	156	178	187
**Larva 15**	228	242	187	187	150	156	178	187
**Larva 16**	214	250	187	193	140	156	187	187
**Larva 17**	N.A.	N.A.	187	187	140	156	178	187
**Larva 18**	N.A.	N.A.	187	187	140	156	178	185
**Larva 19**	N.A.	N.A.	N.A.	N.A.	140	154	178	185
**Larva 20**	N.A.	N.A.	187	187	150	154	178	185
**Larva 21**	N.A.	N.A.	N.A.	N.A.	140	150	185	187
**Larva 22**	N.A.	N.A.	187	187	140	156	178	185
**Larva 23**	N.A.	N.A.	N.A.	N.A.	140	154	178	187
**Larva 24**	N.A.	N.A.	187	193	140	154	187	187
**Larva 25**	N.A.	N.A.	187	187	140	154	187	187
**Larva 26**	N.A.	N.A.	187	193	140	156	N.A.	N.A.
**Larva 27**	N.A.	N.A.	187	187	150	154	N.A.	N.A.
**Larva 28**	N.A.	N.A.	187	193	140	156	N.A.	N.A.
**Larva 29**	N.A.	N.A.	187	193	140	156	N.A.	N.A.
**Larva 30**	N.A.	N.A.	187	187	140	154	N.A.	N.A.
**Larva 31**	N.A.	N.A.	187	193	150	154	187	187
**Larva 32**	N.A.	N.A.	187	187	140	156	178	187
**Larva 33**	N.A.	N.A.	187	187	140	154	178	187
**Larva 34**	N.A.	N.A.	187	193	150	154	178	187
**Larva 35**	N.A.	N.A.	187	193	140	156	178	187
**Larva 36**	N.A.	N.A.	187	187	150	156	185	187
**Larva 37**	N.A.	N.A.	187	187	150	156	185	187
**Larva 38**	N.A.	N.A.	187	193	140	154	185	187
**Larva 39**	N.A.	N.A.	187	193	150	156	178	187
**Larva 40**	N.A.	N.A.	187	187	150	156	187	187

So_10	Locus C.1	Locus C.2	Locus D.1	Locus D.2
**Female**	187	199	140	152
**Male 1**	195	197	140	164
**Male 2**	187	193	150	150
**Larva 01**	197	199	140	152
**Larva 02**	187	197	140	140
**Larva 03**	195	199	140	164
**Larva 04**	187	195	152	164
**Larva 05**	187	197	152	164
**Larva 06**	197	199	140	164
**Larva 07**	187	195	140	140
**Larva 08**	187	195	140	140
**Larva 09**	187	197	140	164
**Larva 10**	197	199	140	164
**Larva 11**	N.A.	N.A.	152	164
**Larva 12**	197	199	140	164
**Larva 13**	197	199	140	164
**Larva 14**	187	197	140	152
**Larva 15**	195	199	140	164
**Larva 16**	195	199	140	152
**Larva 17**	187	197	140	140
**Larva 18**	195	199	140	140
**Larva 19**	187	197	140	140
**Larva 20**	187	197	140	140
**Larva 21**	187	195	140	152
**Larva 22**	197	199	140	140
**Larva 23**	187	195	140	152
**Larva 24**	187	197	N.A.	N.A.
**Larva 25**	N.A.	N.A.	140	140
**Larva 26**	187	195	152	164
**Larva 27**	197	199	140	164
**Larva 28**	187	195	140	152
**Larva 29**	N.A.	N.A.	152	164
**Larva 30**	195	199	140	140
**Larva 31**	187	195	140	152
**Larva 32**	197	199	140	164
**Larva 33**	N.A.	N.A.	140	152
**Larva 34**	195	199	152	164
**Larva 35**	N.A.	N.A.	140	152
**Larva 36**	187	195	152	164
**Larva 37**	N.A.	N.A.	140	140
**Larva 38**	N.A.	N.A.	152	164
**Larva 39**	187	195	152	164
**Larva 40**	187	197	140	152

Table S9. Allele states for tested larva and parents (green) in *Xenos vesparum* (XL_01-10).

XL_01	Locus F.1	Locus F.2
Female	227	231
Male 1	227	227
Male 2	231	233
Larva 01	227	227
Larva 02	231	231
Larva 03	227	227
Larva 04	227	231
Larva 05	227	227
Larva 06	231	231
Larva 07	227	231
Larva 08	227	231
Larva 09	227	227
Larva 10	227	227
Larva 11	227	227
Larva 12	227	227
Larva 13	227	227
Larva 14	227	227
Larva 15	227	227
Larva 16	227	227
Larva 17	227	231
Larva 18	231	231
Larva 19	231	231
Larva 20	227	231
Larva 21	231	231
Larva 22	227	231
Larva 23	227	231
Larva 24	231	231
Larva 25	227	227
Larva 26	227	227
Larva 27	227	231
Larva 28	227	231
Larva 29	231	233
Larva 30	227	227
Larva 31	231	231
Larva 32	227	231
Larva 33	227	227
Larva 34	227	231
Larva 35	227	231
Larva 36	227	227
Larva 37	227	233
Larva 38	231	231
Larva 39	227	231
Larva 40	227	227

XL_02	Locus C.1	LocusC.2	Locus E.1	Locus E.2	LocusF.1	Locus F.2
**Female**	184	188	164	176	227	243
**Male 1**	184	188	164	182	243	243
**Male 2**	182	188	164	170	233	233
**Larva 01**	184	188	164	176	227	243
**Larva 02**	184	188	164	176	227	243
**Larva 03**	184	188	164	176	227	243
**Larva 04**	184	188	164	176	227	243
**Larva 05**	182	188	164	176	N.A.	N.A.
**Larva 06**	184	188	164	176	243	243
**Larva 07**	184	188	176	182	227	243
**Larva 08**	184	188	164	176	227	243
**Larva 09**	N.A.	N.A.	164	176	N.A.	N.A.
**Larva 10**	184	188	164	176	227	243
**Larva 11**	184	188	164	176	227	243
**Larva 12**	184	188	164	176	227	243
**Larva 13**	184	188	164	176	227	243
**Larva 14**	184	188	164	176	227	243
**Larva 15**	184	188	164	176	227	243
**Larva 16**	184	188	164	176	227	243
**Larva 17**	184	188	164	176	227	243
**Larva 18**	184	188	164	176	227	243
**Larva 19**	184	188	164	176	227	243
**Larva 20**	184	188	164	176	227	243
**Larva 21**	184	188	164	176	227	243
**Larva 22**	184	188	164	176	227	243
**Larva 23**	184	188	164	176	227	243
**Larva 24**	182	188	164	176	N.A.	N.A.
**Larva 25**	184	188	164	176	227	243
**Larva 26**	184	188	164	176	227	243
**Larva 27**	184	188	164	176	227	243
**Larva 28**	184	188	164	176	227	243
**Larva 29**	184	188	164	176	227	243
**Larva 30**	184	188	164	176	227	243
**Larva 31**	184	188	164	176	227	243
**Larva 32**	184	188	164	176	227	243
**Larva 33**	184	188	164	176	227	243
**Larva 34**	184	188	164	176	227	243
**Larva 35**	184	188	164	176	227	243
**Larva 36**	184	188	164	176	227	243
**Larva 37**	184	188	164	176	227	243
**Larva 38**	184	188	164	176	227	243
**Larva 39**	184	188	164	176	227	243
**Larva 40**	184	188	164	176	227	243

XL_03	Locus C.1	LocusC.2	Locus E.1	Locus E.2	LocusF.1	Locus F.2
Female	184	188	164	176	233	243
Male 1	186	188	164	170	233	233
Male 2	188	188	164	176	243	243
Larva 01	186	188	164	170	233	233
Larva 02	184	186	164	170	233	243
Larva 03	184	188	164	164	243	243
Larva 04	186	188	164	170	233	233
Larva 05	184	188	164	170	233	243
Larva 06	184	186	164	176	243	243
Larva 07	184	188	170	176	243	243
Larva 08	188	188	170	176	243	243
Larva 09	188	188	170	176	233	233
Larva 10	184	188	164	164	243	243
Larva 11	184	188	170	176	243	243
Larva 12	184	188	164	164	233	233
Larva 13	N.A.	N.A.	164	164	233	233
Larva 14	184	188	164	164	233	233
Larva 15	186	188	164	170	233	233
Larva 16	184	186	170	176	243	243
Larva 17	184	186	164	170	233	233
Larva 18	186	188	164	170	233	243
Larva 19	184	186	164	164	233	243
Larva 20	186	188	164	176	233	233
Larva 21	188	188	170	176	233	233
Larva 22	186	188	170	176	233	233
Larva 23	186	188	170	170	233	233
Larva 24	184	188	170	176	233	233
Larva 25	188	188	164	164	233	243
Larva 26	184	186	164	170	243	243
Larva 27	184	188	164	164	233	233
Larva 28	184	188	164	176	233	233
Larva 29	184	186	164	170	243	243
Larva 30	184	186	164	170	243	243
Larva 31	184	186	164	170	233	233
Larva 32	186	188	170	176	233	243
Larva 33	184	186	164	176	233	243
Larva 34	188	188	164	170	233	233
Larva 35	184	188	164	176	233	233
Larva 36	188	188	164	176	233	243
Larva 37	184	186	170	176	233	233
Larva 38	N.A.	N.A.	164	164	233	233
Larva 39	186	188	164	176	233	233
Larva 40	184	188	164	176	233	233

XL_04	Locus C.1	LocusC.2	Locus E.1	Locus E.2
Female	182	190	164	182
Male 1	188	190	164	164
Male 2	184	186	164	164
Larva 01	182	186	164	164
Larva 02	188	190	164	164
Larva 03	184	190	164	182
Larva 04	N.A.	N.A.	N.A.	N.A.
Larva 05	182	188	164	182
Larva 06	190	190	164	182
Larva 07	182	184	164	164
Larva 08	182	188	164	164
Larva 09	N.A.	N.A.	164	182
Larva 10	184	190	164	164
Larva 11	182	186	164	164
Larva 12	N.A.	N.A.	164	164
Larva 13	186	190	164	182
Larva 14	188	190	164	182
Larva 15	188	190	164	182
Larva 16	186	190	164	164
Larva 17	182	186	164	182
Larva 18	182	190	164	164
Larva 19	184	190	164	182
Larva 20	182	190	164	182
Larva 21	188	190	164	182
Larva 22	182	186	164	164
Larva 23	190	190	164	182
Larva 24	182	188	164	164
Larva 25	184	190	164	164
Larva 26	188	190	164	182
Larva 27	188	190	164	164
Larva 28	184	190	164	182
Larva 29	190	190	164	182
Larva 30	182	188	164	182
Larva 31	190	190	164	182
Larva 32	188	190	164	164
Larva 33	182	184	164	182
Larva 34	188	190	164	182
Larva 35	186	190	164	164
Larva 36	182	188	164	182
Larva 37	186	190	164	182
Larva 38	182	184	164	164
Larva 39	188	190	164	182
Larva 40	N.A.	N.A.	164	182

XL_05	Locus C.1	LocusC.2	Locus F.1	Locus F.2	LocusG.1	Locus G.2
**Female**	186	188	233	243	123	133
**Male 1**	188	188	233	233	123	137
**Male 2**	186	188	243	243	123	123
**Larva 01**	186	188	243	243	N.A.	N.A.
**Larva 02**	186	188	243	243	N.A.	N.A.
**Larva 03**	188	188	233	233	N.A.	N.A.
**Larva 04**	186	188	233	233	N.A.	N.A.
**Larva 05**	188	188	233	233	N.A.	N.A.
**Larva 06**	186	188	233	243	N.A.	N.A.
**Larva 07**	186	188	233	233	N.A.	N.A.
**Larva 08**	186	188	N.A.	N.A.	N.A.	N.A.
**Larva 09**	186	188	243	243	133	137
**Larva 10**	186	188	233	233	N.A.	N.A.
**Larva 11**	186	188	233	243	N.A.	N.A.
**Larva 12**	188	188	233	233	N.A.	N.A.
**Larva 13**	188	188	233	233	123	137
**Larva 14**	188	188	243	243	N.A.	N.A.
**Larva 15**	186	188	243	243	N.A.	N.A.
**Larva 16**	186	188	233	243	N.A.	N.A.
**Larva 17**	186	188	243	243	N.A.	N.A.
**Larva 18**	186	188	233	233	133	137
**Larva 19**	186	188	233	233	N.A.	N.A.
**Larva 20**	186	186	243	243	N.A.	N.A.
**Larva 21**	188	188	233	233	N.A.	N.A.
**Larva 22**	186	188	233	233	133	137
**Larva 23**	186	188	233	243	N.A.	N.A.
**Larva 24**	186	188	233	233	N.A.	N.A.
**Larva 25**	N.A.	N.A.	243	243	N.A.	N.A.
**Larva 26**	188	188	233	233	123	137
**Larva 27**	186	188	233	243	N.A.	N.A.
**Larva 28**	186	186	243	243	N.A.	N.A.
**Larva 29**	188	188	243	243	N.A.	N.A.
**Larva 30**	186	188	243	243	N.A.	N.A.
**Larva 31**	186	188	243	243	N.A.	N.A.
**Larva 32**	188	188	233	233	N.A.	N.A.
**Larva 33**	188	188	243	243	N.A.	N.A.
**Larva 34**	188	188	233	233	N.A.	N.A.
**Larva 35**	186	188	243	243	N.A.	N.A.
**Larva 36**	188	188	233	243	N.A.	N.A.
**Larva 37**	188	188	243	243	N.A.	N.A.
**Larva 38**	188	188	243	243	N.A.	N.A.
**Larva 39**	188	188	243	243	N.A.	N.A.
**Larva 40**	188	188	233	233	N.A.	N.A.

XL_06	Locus C.1	LocusC.2	Locus E.1	Locus E.2	Locus G.1	Locus G.2
**Female**	188	190	176	182	133	133
**Male 1**	186	186	164	174	133	133
**Male 2**	184	188	174	176	133	135
**Larva 01**	188	190	176	N.A.	N.A.	N.A.
**Larva 02**	188	188	174	176	N.A.	N.A.
**Larva 03**	186	188	174	182	N.A.	N.A.
**Larva 04**	186	188	164	176	N.A.	N.A.
**Larva 05**	186	190	174	182	N.A.	N.A.
**Larva 06**	188	188	174	182	N.A.	N.A.
**Larva 07**	186	188	164	176	N.A.	N.A.
**Larva 08**	186	188	164	182	N.A.	N.A.
**Larva 09**	184	190	176	176	135	135
**Larva 10**	186	190	174	176	N.A.	N.A.
**Larva 11**	184	190	176	182	135	135
**Larva 12**	186	188	164	176	N.A.	N.A.
**Larva 13**	188	188	174	182	N.A.	N.A.
**Larva 14**	186	188	174	176	N.A.	N.A.
**Larva 15**	188	188	176	176	N.A.	N.A.
**Larva 16**	186	190	164	176	N.A.	N.A.
**Larva 17**	186	190	174	182	N.A.	N.A.
**Larva 18**	184	188	174	182	135	135
**Larva 19**	186	188	174	182	N.A.	N.A.
**Larva 20**	186	190	174	176	N.A.	N.A.
**Larva 21**	186	188	174	176	N.A.	N.A.
**Larva 22**	184	188	174	182	135	135
**Larva 23**	186	190	164	182	N.A.	N.A.
**Larva 24**	186	190	164	182	N.A.	N.A.
**Larva 25**	188	190	176	182	N.A.	N.A.
**Larva 26**	186	188	164	182	N.A.	N.A.
**Larva 27**	188	190	174	182	N.A.	N.A.
**Larva 28**	184	190	174	182	N.A.	N.A.
**Larva 29**	188	188	174	176	N.A.	N.A.
**Larva 30**	186	188	174	182	N.A.	N.A.
**Larva 31**	188	188	176	176	N.A.	N.A.
**Larva 32**	186	188	164	182	N.A.	N.A.
**Larva 33**	186	190	174	182	N.A.	N.A.
**Larva 34**	188	190	174	176	N.A.	N.A.
**Larva 35**	184	188	174	182	135	135
**Larva 36**	186	188	164	176	N.A.	N.A.
**Larva 37**	184	188	174	176	135	135
**Larva 38**	186	190	164	182	N.A.	N.A.
**Larva 39**	186	190	174	182	N.A.	N.A.
**Larva 40**	188	188	174	176	N.A.	N.A.

XL_07	Locus A.1	Locus A.2	Locus E.1	Locus E.2	Locus F.1	Locus F.2
**Female**	156	164	166	176	233	233
**Male 1**	164	168	174	174	233	233
**Male 2**	156	164	164	164	241	241
**Larva 01**	156	164	164	176	233	241
**Larva 02**	164	164	164	166	233	233
**Larva 03**	164	164	164	166	233	241
**Larva 04**	164	164	164	166	233	241
**Larva 05**	164	164	164	166	233	233
**Larva 06**	156	164	164	166	233	233
**Larva 07**	156	164	164	176	233	233
**Larva 08**	156	164	164	176	233	233
**Larva 09**	164	164	164	166	233	233
**Larva 10**	164	164	164	176	233	241
**Larva 11**	164	164	164	166	233	233
**Larva 12**	164	164	164	166	233	233
**Larva 13**	164	164	164	166	233	233
**Larva 14**	156	164	164	176	233	233
**Larva 15**	164	164	164	166	233	233
**Larva 16**	156	164	164	166	233	233
**Larva 17**	156	164	164	166	233	241
**Larva 18**	164	164	164	166	233	233
**Larva 19**	156	164	164	176	233	233
**Larva 20**	156	164	164	176	233	233
**Larva 21**	156	164	164	166	233	233
**Larva 22**	156	164	164	166	233	233
**Larva 23**	164	164	164	166	233	233
**Larva 24**	156	164	164	176	233	241
**Larva 25**	164	164	164	176	233	233
**Larva 26**	164	164	164	166	233	241
**Larva 27**	156	164	164	176	233	241
**Larva 28**	164	164	164	166	N.A.	N.A.
**Larva 29**	164	164	164	176	233	233
**Larva 30**	164	164	164	166	233	233
**Larva 31**	164	164	164	166	233	233
**Larva 32**	164	164	164	176	233	241
**Larva 33**	156	164	164	176	233	241
**Larva 34**	156	164	164	176	233	233
**Larva 35**	156	164	164	176	233	233
**Larva 36**	164	164	164	166	233	233
**Larva 37**	156	164	164	166	233	233
**Larva 38**	N.A.	N.A.	164	166	N.A.	N.A.
**Larva 39**	156	164	164	176	233	233
**Larva 40**	164	164	164	166	233	233

XL_08	Locus D.1	Locus D.2	Locus F.1	Locus F.2	Locus G.1	Locus G.2
**Female**	141	139	233	241	127	133
**Male 1**	131	149	233	233	123	127
**Male 2**	133	139	233	235	127	127
**Larva 01**	131	141	233	233	127	133
**Larva 02**	131	139	233	241	127	133
**Larva 03**	139	149	233	241	123	133
**Larva 04**	139	149	233	241	127	133
**Larva 05**	131	139	233	233	123	133
**Larva 06**	131	141	233	233	127	133
**Larva 07**	131	141	233	233	123	133
**Larva 08**	139	149	233	233	127	133
**Larva 09**	133	139	233	241	133	133
**Larva 10**	141	149	241	241	123	133
**Larva 11**	131	139	233	241	127	127
**Larva 12**	131	141	233	241	123	133
**Larva 13**	131	141	233	241	127	133
**Larva 14**	131	141	233	241	123	133
**Larva 15**	141	149	233	241	123	133
**Larva 16**	139	149	233	241	123	133
**Larva 17**	131	139	233	233	127	127
**Larva 18**	131	139	233	233	123	133
**Larva 19**	131	139	N.A.	N.A.	123	133
**Larva 20**	131	141	233	241	123	133
**Larva 21**	139	141	233	241	127	133
**Larva 22**	141	149	233	241	127	133
**Larva 23**	133	141	233	241	127	133
**Larva 24**	131	141	233	241	127	133
**Larva 25**	141	149	N.A.	N.A.	127	133
**Larva 26**	141	149	233	241	127	133
**Larva 27**	131	139	233	241	123	133
**Larva 28**	139	149	233	233	127	133
**Larva 29**	139	149	N.A.	N.A.	127	133
**Larva 30**	139	149	233	233	123	133
**Larva 31**	131	139	233	233	127	133
**Larva 32**	139	149	241	241	123	133
**Larva 33**	131	141	233	233	123	133
**Larva 34**	131	141	233	241	127	133
**Larva 35**	139	149	233	241	123	133
**Larva 36**	131	141	233	233	123	133
**Larva 37**	131	139	233	233	123	133
**Larva 38**	141	149	233	233	127	133
**Larva 39**	131	141	233	233	127	127
**Larva 40**	131	141	233	233	127	133

XL_09	Locus D.1	Locus D.2	Locus E.1	Locus E.2	Locus F.1	Locus F.2
**Female**	133	151	170	178	233	233
**Male 1**	139	151	164	164	233	233
**Male 2**	133	133	164	170	237	239
**Larva 01**	139	151	164	178	233	233
**Larva 02**	133	139	164	170	233	233
**Larva 03**	139	151	164	178	233	233
**Larva 04**	151	151	164	178	N.A.	N.A.
**Larva 05**	139	151	164	178	N.A.	N.A.
**Larva 06**	133	139	164	170	233	233
**Larva 07**	133	151	164	170	233	233
**Larva 08**	133	151	N.A.	N.A.	N.A.	N.A.
**Larva 09**	133	151	170	178	N.A.	N.A.
**Larva 10**	151	151	164	178	233	233
**Larva 11**	133	151	164	170	N.A.	N.A.
**Larva 12**	133	151	164	170	N.A.	N.A.
**Larva 13**	133	139	N.A.	N.A.	N.A.	N.A.
**Larva 14**	139	151	164	170	233	233
**Larva 15**	139	151	164	178	233	233
**Larva 16**	139	151	164	178	233	233
**Larva 17**	N.A.	N.A.	164	170	N.A.	N.A.
**Larva 18**	133	151	164	170	N.A.	N.A.
**Larva 19**	133	139	164	170	N.A.	N.A.
**Larva 20**	133	133	164	170	N.A.	N.A.
**Larva 21**	133	151	164	170	233	233
**Larva 22**	151	151	164	178	233	233
**Larva 23**	139	151	164	178	233	233
**Larva 24**	133	151	164	170	N.A.	N.A.
**Larva 25**	133	133	170	170	N.A.	N.A.
**Larva 26**	133	151	170	178	N.A.	N.A.
**Larva 27**	133	151	164	170	N.A.	N.A.
**Larva 28**	133	151	164	170	233	233
**Larva 29**	133	139	164	170	233	233
**Larva 30**	151	151	164	178	233	233
**Larva 31**	151	151	164	178	233	233
**Larva 32**	139	151	164	178	233	233
**Larva 33**	133	151	164	170	233	233
**Larva 34**	133	151	164	170	N.A.	N.A.
**Larva 35**	133	151	164	170	N.A.	N.A.
**Larva 36**	133	139	164	170	233	233
**Larva 37**	133	139	164	170	233	233
**Larva 38**	133	151	164	170	233	233
**Larva 39**	133	151	164	170	233	233
**Larva 40**	151	151	164	178	233	233

XL_10	Locus A.1	Locus A.2	Locus D.1	Locus D.2	Locus F.1	Locus F.2
Female	156	168	139	143	227	233
Male 1	164	168	131	139	243	243
Male 2	164	168	133	149	233	233
Larva 01	156	164	133	143	233	233
Larva 02	164	168	133	139	233	233
Larva 03	156	164	143	149	233	233
Larva 04	164	168	139	149	233	233
Larva 05	156	164	143	149	227	227
Larva 06	156	164	133	143	233	233
Larva 07	164	168	139	149	227	233
Larva 08	156	164	143	149	233	233
Larva 09	164	168	N.A.	N.A.	227	233
Larva 10	156	164	143	149	227	227
Larva 11	164	168	139	149	233	233
Larva 12	156	164	133	143	227	227
Larva 13	164	168	133	139	227	227
Larva 14	164	168	139	149	233	233
Larva 15	164	168	139	149	233	233
Larva 16	164	168	139	149	227	227
Larva 17	164	168	139	149	227	233
Larva 18	164	168	139	149	233	233
Larva 19	164	168	133	139	227	233
Larva 20	164	168	139	149	233	233
Larva 21	164	168	133	143	233	233
Larva 22	164	168	133	139	227	227
Larva 23	156	164	143	149	227	227
Larva 24	156	164	143	149	233	233
Larva 25	156	164	133	143	227	233
Larva 26	156	164	133	143	233	233
Larva 27	156	164	133	143	227	233
Larva 28	156	164	143	149	233	233
Larva 29	156	164	133	143	233	233
Larva 30	156	164	133	143	227	233
Larva 31	164	168	133	139	227	227
Larva 32	156	164	143	149	233	233
Larva 33	156	164	143	149	233	233
Larva 34	156	164	133	143	227	227
Larva 35	164	168	133	139	233	233
Larva 36	164	164	139	143	227	233
Larva 37	156	164	133	143	227	233
Larva 38	164	168	133	139	227	233
Larva 39	164	168	133	139	227	227
Larva 40	164	168	139	149	233	233
